# Tumor-derived IL-6 and trans-signaling among tumor, fat, and muscle mediate pancreatic cancer cachexia

**DOI:** 10.1084/jem.20190450

**Published:** 2021-04-14

**Authors:** Joseph E. Rupert, Ashok Narasimhan, Daenique H.A. Jengelley, Yanlin Jiang, Jianguo Liu, Ernie Au, Libbie M. Silverman, George Sandusky, Andrea Bonetto, Sha Cao, Xiaoyu Lu, Thomas M. O’Connell, Yunlong Liu, Leonidas G. Koniaris, Teresa A. Zimmers

**Affiliations:** 1 Department of Biochemistry, Indiana University School of Medicine, Indianapolis, IN; 2 Department of Surgery, Indiana University School of Medicine, Indianapolis, IN; 3 Indiana University Simon Comprehensive Cancer Center, Indianapolis, IN; 4 Department of Pathology, Indiana University School of Medicine, Indianapolis, IN; 5 Department of Otolaryngology–Head and Neck Surgery, Indiana University School of Medicine, Indianapolis, IN; 6 Department of Anatomy, Cell Biology and Physiology, Indiana University School of Medicine, Indianapolis, IN; 7 Indiana Center for Musculoskeletal Health, Indiana University School of Medicine, Indianapolis, IN; 8 Department of Biostatistics, Indiana University School of Medicine, Indianapolis, IN; 9 Department of Molecular and Medical Genetics, Indiana University School of Medicine, Indianapolis, IN; 10 Center for Computational Biology and Bioinformatics, Indiana University School of Medicine, Indianapolis, IN; 11 Richard L. Roudebush Veterans Administration Medical Center, Indianapolis, IN

## Abstract

Most patients with pancreatic adenocarcinoma (PDAC) suffer cachexia; some do not. To model heterogeneity, we used patient-derived orthotopic xenografts. These phenocopied donor weight loss. Furthermore, muscle wasting correlated with mortality and murine IL-6, and human IL-6 associated with the greatest murine cachexia. In cell culture and mice, PDAC cells elicited adipocyte IL-6 expression and IL-6 plus IL-6 receptor (IL6R) in myocytes and blood. PDAC induced adipocyte lipolysis and muscle steatosis, dysmetabolism, and wasting. Depletion of IL-6 from malignant cells halved adipose wasting and abolished myosteatosis, dysmetabolism, and atrophy. In culture, adipocyte lipolysis required soluble (s)IL6R, while IL-6, sIL6R, or palmitate induced myotube atrophy. PDAC cells activated adipocytes to induce myotube wasting and activated myotubes to induce adipocyte lipolysis. Thus, PDAC cachexia results from tissue crosstalk via a feed-forward, IL-6 trans-signaling loop. Malignant cells signal via IL-6 to muscle and fat, muscle to fat via sIL6R, and fat to muscle via lipids and IL-6, all targetable mechanisms for treatment of cachexia.

## Teaser

Cachexia increases morbidity and mortality. Rupert et al. show PDAC cachexia results from tissue crosstalk via an IL-6 trans-signaling loop. Malignant cells signal via IL-6 to muscle and fat, muscle to fat via sIL6R, and fat to muscle via lipids and IL-6.

## Introduction

Pancreatic ductal adenocarcinoma (PDAC) is among the deadliest cancers, with a 5-yr mortality rate of >91% (Siegel et al., 2019). Cachexia, the involuntary loss of fat, muscle, and bone mass, affects over 80% of patients with PDAC and leads to increased morbidity and mortality (Hendifar et al., 2018; Sun et al., 2015; von Haehling et al., 2016). Both cancer and cachexia are associated with systemic inflammation affecting multiple organ systems (Argilés et al., 2019; Onesti and Guttridge, 2014). While various cytokines, chemokines, and growth factors are changed in PDAC, IL-6 specifically has been positively correlated with PDAC presence (Holmer et al., 2014), disease progression (Ramsey et al., 2019), mortality (Babic et al., 2018; Suh et al., 2013), and cachexia (Okada et al., 1998; Ebrahimi et al., 2004; Martignoni et al., 2005). Although circulating IL-6 levels are not always detectable in early PDAC nor always correlated with cachexia severity (Ramsey et al., 2019; Talbert et al., 2018), higher tumor staining for IL-6 is associated with PDAC cachexia (Martignoni et al., 2005) and induction of monocyte IL-6 is predictive of survival in PDAC (Moses et al., 2009), suggesting that the serum levels of this short-lived cytokine might not be an appropriate measure of tissue activity. Functional data also support a role for IL-6 in PDAC tumor development (Lesina et al., 2011), progression (Zhang et al., 2013), metastasis (Razidlo et al., 2018), antitumor immunity (Flint et al., 2016), and response to chemotherapy (Long et al., 2017). IL-6 levels are high in PDAC models with weight loss (Flint et al., 2016), and IL-6 is functionally linked to cachexia in murine C26 colon adenocarcinoma and other models of cancer cachexia (Baltgalvis et al., 2008; Bonetto et al., 2012; Bonetto et al., 2011; Narsale and Carson, 2014). Moreover, IL-6 is sufficient to induce cachexia in mice (Baltgalvis et al., 2009; Chen et al., 2016; Tsujinaka et al., 1996) as well as lipolysis and atrophy in cultured adipocytes (Trujillo et al., 2004) and myotubes (Bonetto et al., 2012), respectively. IL-6 can be both detrimental and beneficial. While chronically increased IL-6 is associated with insulin resistance, inflammation, adipose tissue lipolysis, and muscle wasting in diseases from cancer and obesity to sepsis and burn injury (Kraakman et al., 2015; Pedroso et al., 2012; van Hall, 2012), acute expression of IL-6 promotes liver regeneration after injury (Jin et al., 2006; Koniaris et al., 2003) and is required for muscle regeneration, exercise-induced hypertrophy, and recovery from disuse atrophy (Begue et al., 2013; McKay et al., 2009; Washington et al., 2011).

IL-6 initiates signal transduction by first binding to either the membrane-bound form of the IL-6 receptor (IL6R), also known as glycoprotein 80 (GP80), or its soluble form (sIL6R; Schaper and Rose-John, 2015). Proteolytic shedding of a 55-kD fragment in tissues expressing membrane IL6R results in circulating sIL6R (Schaper and Rose-John, 2015), an activity mediated in part through intracellular accumulation of phorbol esters and activation of protein kinase C θ (PKCθ; Müllberg et al., 1992). Both complexes of IL-6 with membrane or sIL6R bind the ubiquitously expressed membrane coreceptor IL-6 signal transducer, also known as glycoprotein 130 (GP130). The activity elicited by IL-6 and membrane IL6R is considered classical or cis signaling, while activity instigated by IL-6 with sIL6R is known as trans-signaling. Formation of either complex leads to trans-phosphorylation and activation of JAKs, which phosphorylate the transcription factor STAT3, promoting STAT3 dimerization and translocation to the nucleus (Taniguchi and Karin, 2014). Unlike GP130, IL6R expression is not ubiquitous across cell types; thus, sIL6R trans-signaling allows for IL-6 signaling in IL6R-negative cells (Rosean et al., 2014). Signaling through the membrane receptor is largely beneficial, while trans-signaling is generally pathological (Schaper and Rose-John, 2015). A growing number of studies suggest that neutralization of IL6R could have greater utility versus targeting IL-6 directly (Kraakman et al., 2015).

Given its various roles in PDAC, lipolysis, and muscle wasting, here we investigated the effects of IL-6 emanating from PDAC tumor cells on tissue crosstalk and cachexia using patient-derived orthotopic xenografts and mouse PDAC cells deleted for IL-6 (KPC IL6^KO^) in a mouse model of pancreatic cancer cachexia. Furthermore, we used in vitro media swap studies to characterize tumor, fat, and muscle crosstalk via IL6R trans-signaling in PDAC cachexia. Our results suggest a feed-forward signaling loop between tumor-derived IL-6, skeletal muscle steatosis and production of sIL6R, and adipose tissue IL-6 production working in concert to exacerbate skeletal muscle wasting in PDAC.

## Results

### High cachexia potential of human tumors associates with IL-6, and malignant cells express IL-6 in a fraction of tumors

**Figure 1.**
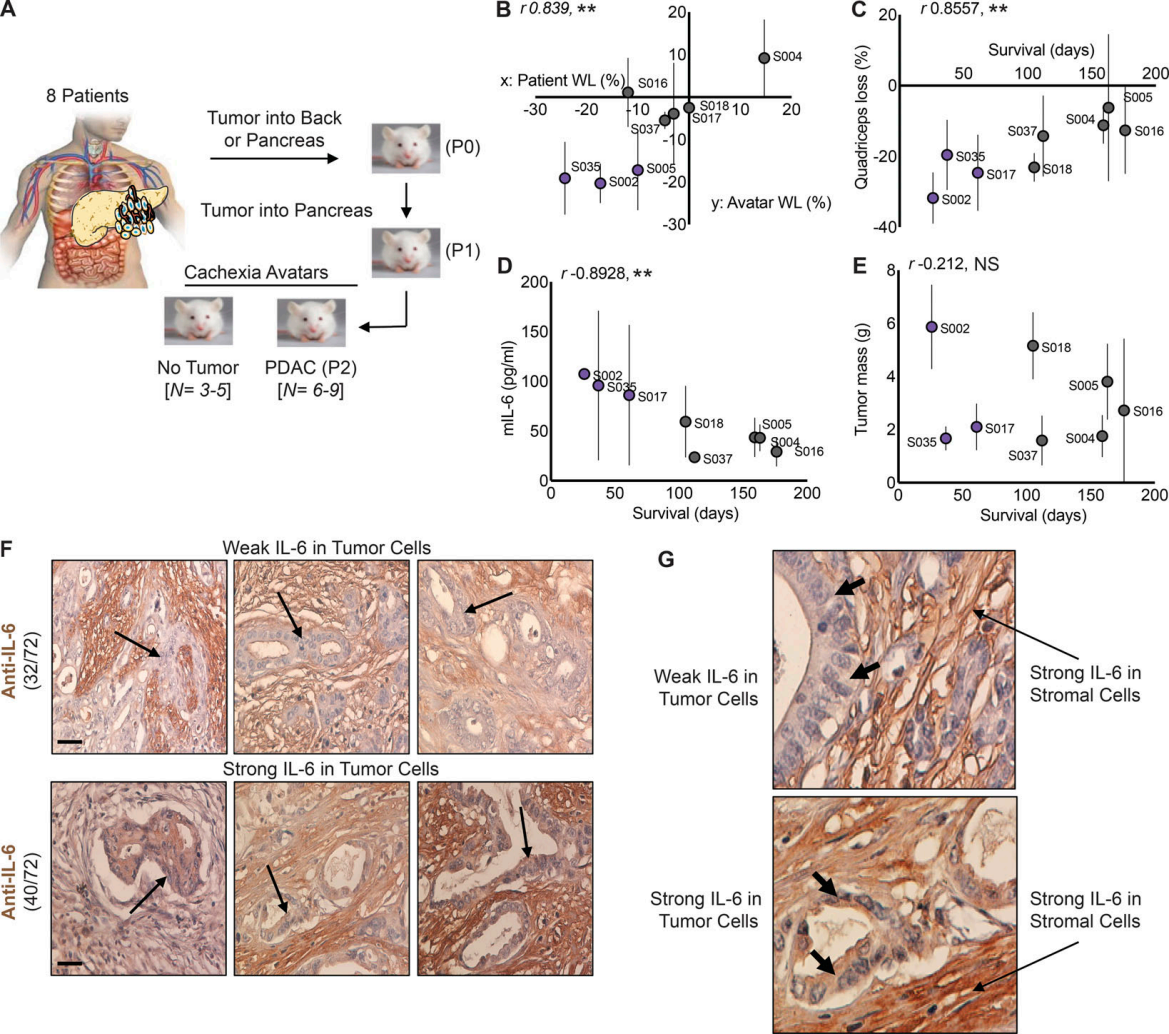
**IL-6 protein expression is associated with increased cachexia severity and mortality in a mouse xenograft model of human PDAC. (A)** Experimental outline for the generation of the mouse xenograft model (PDX) of human PDAC. **(B)** Changes in body weight of human patients with PDAC and their corresponding xenograft mouse avatar. **(C–E)** Implantation of human tumors into mice shows correlations between muscle loss and mortality (C), mIL-6 and mortality (D), and mIL-6 and muscle loss (E). Mice with the highest levels of mIL-6 also had increased human IL-6 (D;****S002, S035, S017). Data shown are mean ± SD, where *n* = 6–9 mouse avatars per patient tumor. Statistical differences (**, P < 0.005) and *r* values were determined using Pearson correlation coefficient analysis. **(F and G)** Human PDAC tumors obtained from US Biomax were reacted for IL-6 using IHC. **(F)** Tumor sections were classified as having either high or low expression of IL-6 specifically in tumor epithelial cells (arrows). Of the 72 tumors, 40 had low tumor cell IL-6 expression, and 32 had high tumor cell IL-6 expression. **(G)** Increased magnification to show PDAC tumor cell IL-6 expression, with arrows added to show tumor cells with weak IL-6 expression (top) and strong IL-6 expression (bottom). Scale bar = 40 µm. WL, weight loss.

Patients with PDAC exhibit heterogeneity in cachexia presentation, with the majority of patients suffering severe cachexia, but some 15% of patients experiencing no cachexia at all. To model this heterogeneity, we generated patient-derived PDAC orthotopic xenograft mouse models (PDXs) by implanting pieces of human tumor into a recipient NSG: NOD.Cg-*Prkdcscid Il2rgtm1Wjl*/SzJ mouse (passage [P] 0), then passaging tumor fragments into a subsequent host (P1), and finally characterizing the cachexia phenotype in *n* = 8–10 mice in P2 versus sham controls (Fig. 1 A). We developed eight such cachexia avatar models. At the third passage, malignant cells but not stromal cells should be of patient origin. These PDX tumors showed a range of capacity to induce cachexia in mice. Strikingly, the severity of cachexia in the xenografted mice at euthanasia correlated strongly to presurgery 6-mo history of weight loss in the donors (Fig. 1 B). Loss of quadriceps mass (Fig. 1 C) and circulating levels of murine IL-6 (mIL-6) correlated negatively with survival (Fig. 1 D), although tumor mass did not (Fig. 1 E). Most interestingly, the three avatar groups with the shortest survival, highest muscle loss, and highest mIL-6 levels demonstrated detectable human IL-6 in blood (Fig. 1, B–E, purple dots; and Table 1). Overall, these results indicate that (1) PDAC tumors demonstrate heterogeneity in the ability to induce cachexia in the host; (2) this characteristic can be modeled in mice; and (3) the capacity to induce cachexia is in part tumor-intrinsic and (4) is partly due to IL-6 production from malignant cells. Thus, PDAC epithelial cells are a source of IL-6 in both the tumor micro- and macro-environment and apparently significantly contribute to cachexia and survival.

**Table 1.** Tumor donor patient characteristics for cachexia avatar lines

ID	Age (yr)	Sex	TMN staging^a^	CA19.9	BMI (kg/m^2^)	6M WL	Avatar hIL-6 (pg/ml)
S002	84	M	T3, N1, Mx	97	29.6	−17.3%	>700
S004	84	F	T3, N1	200	27.3	14.7%	-
S005	70	F	T1, N1	103	26.9	−10.0%	-
S016	75	M	T3, N1	2,252	21.7	−11.9%	-
S017	69	M	T3, N1	25	25.6	−3.2%	1.94 ± 0.95
S018	68	M	T3, N1, Mx	544	26.6	−7.2%	-
S035	65	M	T2, N1	61	49.1	−30.6%	513.9 ± 152.9
S037	82	F	T3, N0, Mx	11	27	−4.7%	-

BMI, body mass index; F, female; hIL, human IL; M, male; 6M WL, 6-mo weight loss.

a Pathological Tumor Node Metastasis Staging: T1 in pancreas with tumor <2 cm; T2 in pancreas and 2 cm < tumor < 4 cm; T3, beyond pancreas and tumor >4 cm. N0, no regional lymph nodes involved; N1, 1–3 regional lymph nodes involved; Mx, metastasis cannot be measured.

**Figure S1.**
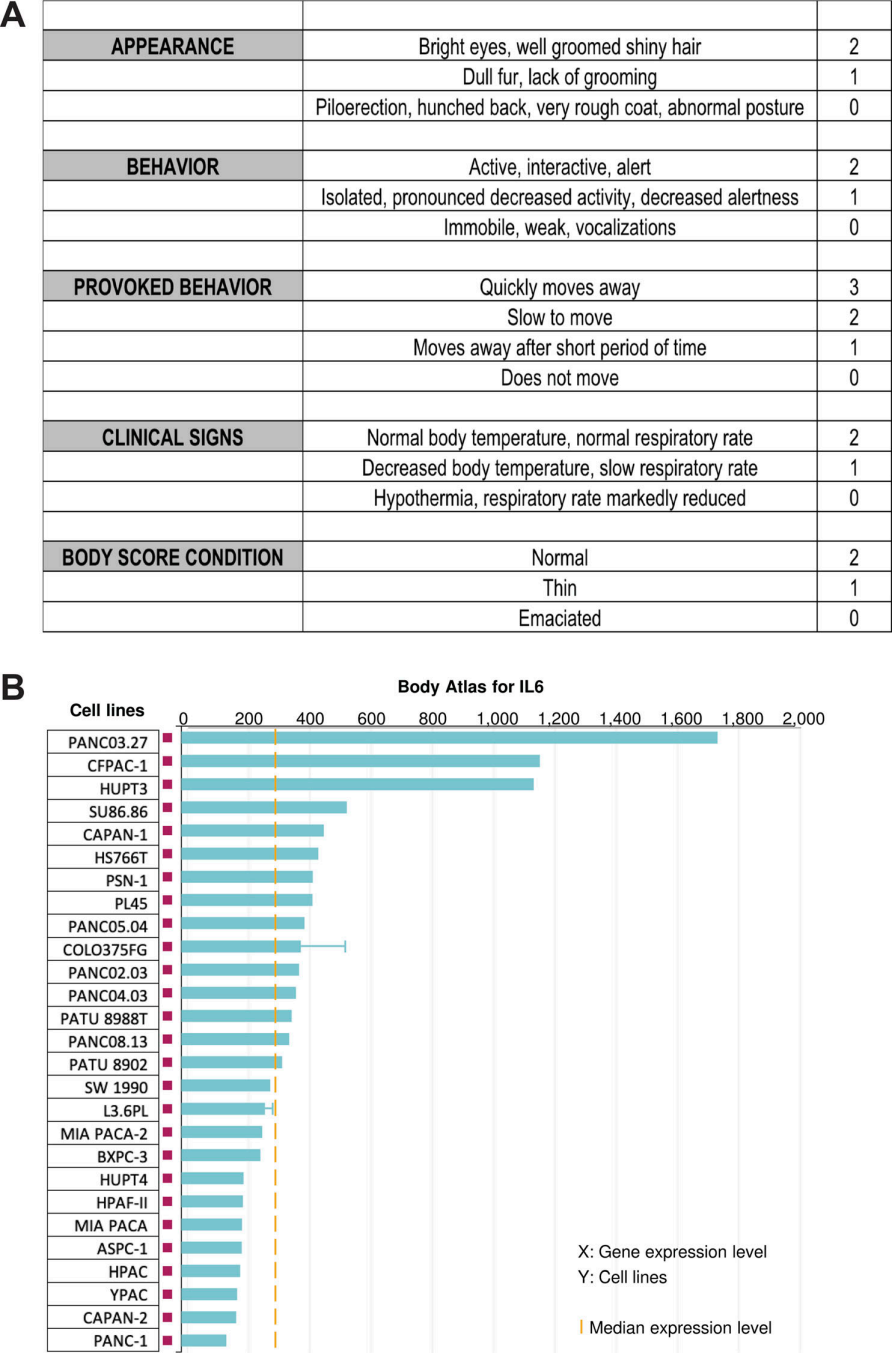
**Body condition scoring for cachexia avatar studies in ****Fig. 1****, as described. (A)** Mice were euthanized when a majority reached a total score of 2. **(B)** Expression levels of *IL6* mRNA across multiple human pancreatic cancer cell lines from Illumina BaseSpace Correlation Engine illustrating heterogeneity in *IL6* expression. Blue bar indicates median *Il6* expression for each cell line; yellow line indicates median *Il6* expression for the entire group; error bars indicate SD where applicable.

To investigate the frequency with which tumors express IL-6 from malignant cells, we performed immunohistochemistry (IHC) on a PDAC tumor microarray. Stromal IL-6 staining was observed in all tumors tested (Fig. 1 F) as previously reported (Mace et al., 2018). However, IL-6 staining in tumor epithelial cells was also observed in ∼44% of tumors. Of 72 tumor samples, 32 demonstrated weak staining in tumor epithelial cells (Fig. 1, F and G), and 40 samples had strong staining intensity (Fig. 1, F and G). Expression profiling also confirmed a range of *IL*-*6* expression from high (PSN-1, Panc03.27, PANC-1) to low (MIA PaCA, AsPC-1, HuPT4) in human and murine PDAC cell lines (Fig. S1). Taken with the avatar data, these results suggest that IL-6 production from malignant cells might describe a subgroup of highly cachexia-inducing tumors.

### PDAC-induced cachexia and mortality are significantly improved by deletion of IL-6 from tumor cells

**Figure 2.**
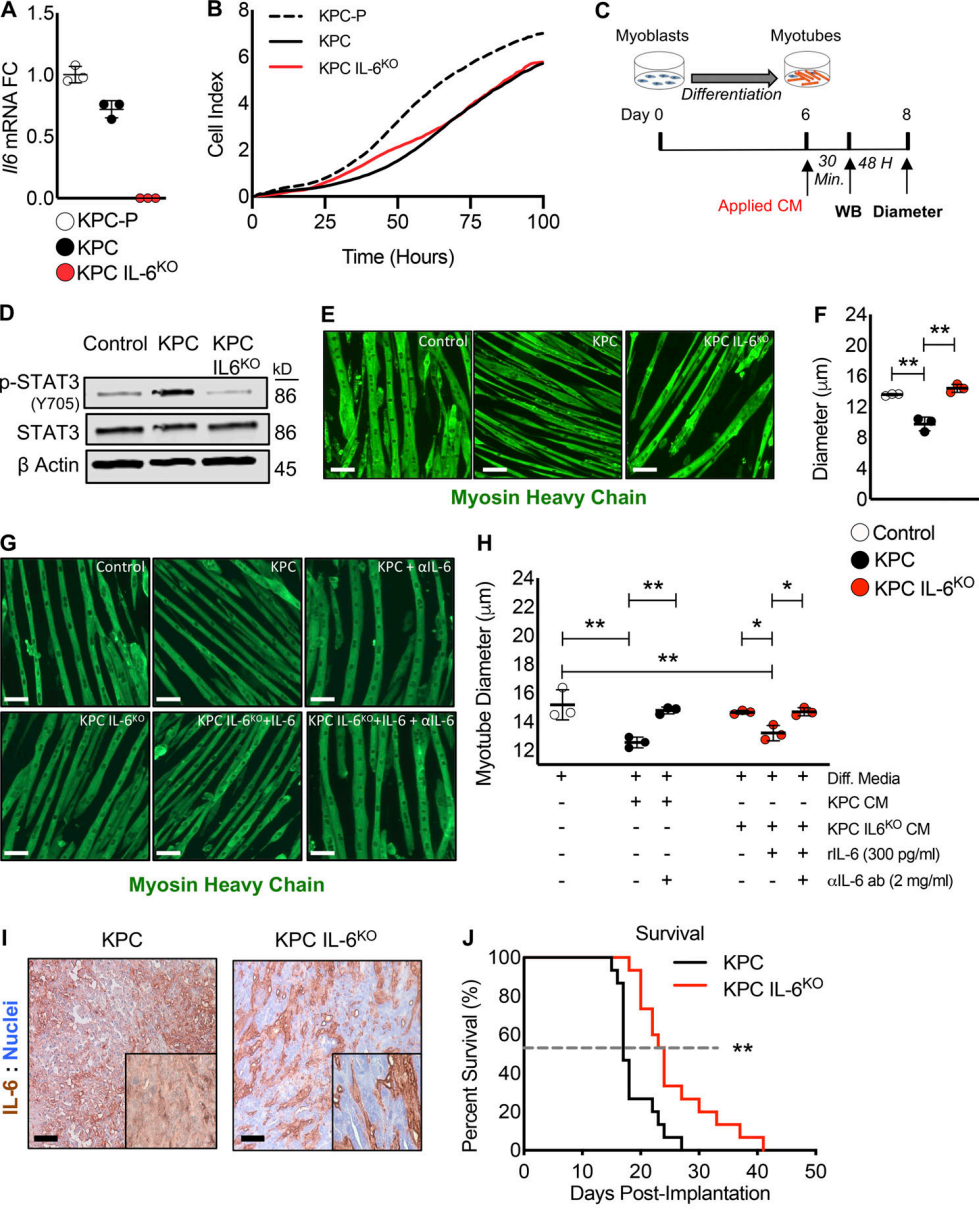
**Deletion of IL-6 from KPC cells prevented muscle wasting in vitro and increased survival in mice. (A)** Targeted mutagenesis of the *Il6 *gene was performed, and a transfection control clone (KPC) and an *Il6 *ablated clone (KPC IL6^KO^) were selected for use in downstream experiments based on their *Il6* expression relative to the untransfected parental cell line (KPC-P). Clones were cultured individually in triplicate for *n* = 3 per group, and error bars are mean plus SD. **(B)** To determine if deletion of IL-6 affected tumor cell growth, a proliferation assay was performed comparing the clones, where clones were cultured in triplicate (*n* = 3 per group) and measurements were taken every hour for 100 h; growth curves represent the mean proliferation of triplicate wells over time. **(C) **Myoblasts were differentiated into myotubes and treated in triplicate (*n* = 3 per group) with 30% CM from tumor clones to measure effects on myotube atrophy and the activation of STAT3. **(D)** Western blotting (WB) analysis using the pooled myotube protein lysates from *n* = 3 per group was performed to measure phosphorylation of STAT3 (p-STAT3) as an indication of STAT3 activation. **(E and F)** Myotubes were visualized using IF with an anti-myosin heavy chain (MHC) antibody (E), and myotube atrophy was measured from 20 random fields per well (n∼150 myotube diameter measurements per well, *n* = 3 wells per group). Scale bar = 50 µm. Error bars represent mean myotube diameter and SD, and significant differences (**, P < 0.001) were determined using ANOVA and Tukey’s multiple comparisons test (F). **(G and H)** To verify atrophy was influenced by IL-6, myotubes were treated in triplicate with KPC CM and IL-6 neutralizing antibody as well as KPC IL6^KO^ CM plus recombinant IL-6 with and without the presence of an anti–IL-6 neutralizing antibody. Myotubes were visualized with MHC IF, and diameter was measured as described in F. Scale bar = 50 µm. Error bars are mean and SD, and significant differences (*, P < 0.05; **, P < 0.001) were determined using ANOVA and Tukey’s multiple comparisons test. **(I)** KPC tumor cells were orthotopically implanted into mice, and tumors were excised, sectioned, and reacted with anti–IL-6 IHC to verify tumor cell IL-6 deletion. Insets are increased magnification to show staining; scale bar = 20 µm. **(J)** Survival was measured in mice orthotopically implanted with KPC and KPC IL6^KO^ tumor cells. The dashed line represents median survival, *n* = 15 per group, and statistical difference (**, P < 0.001) was determined using a Kaplan-Meier estimate. All panels represent data from single experiments. Diff. media, DM.

**Figure S2.**
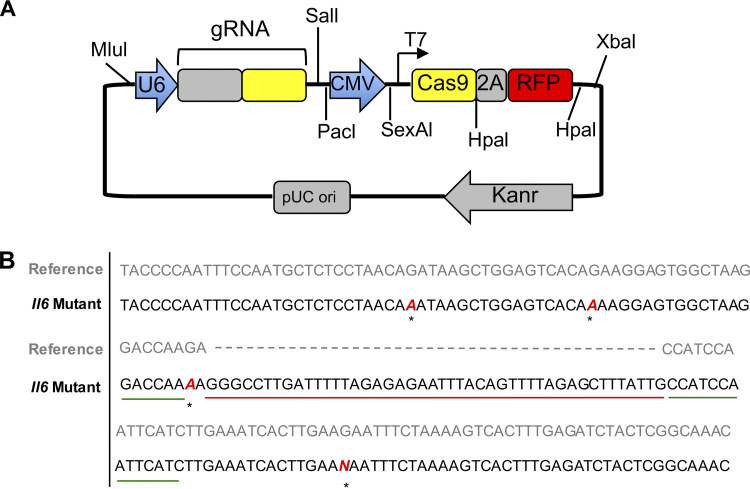
**Generation of KPC IL6^KO^ cells.** **(A) **CRISPR gRNA plasmid map. **(B)** DNA sequencing was performed at the CRISPR/Cas9 target site (green line) within the *mIl6* gene sequence, showing an insertion mutation of 45 nucleotides (red line) into the KPC IL6^KO^ mutant sequence. Asterisks indicate point mutations.

To evaluate the effects of tumor cell–derived IL-6 in PDAC cachexia, we used CRISPR/Cas9 to edit the *Il6* gene in a cell line isolated from the KPC (genetically engineered mouse model, Kras^G12D^;Trp53^R172H^:Pdx1-Cre). A similarly transfected clone without guide RNAs (gRNAs) was used as a control (KPC). *Il6* mRNA was detected in the untransfected parental KPC cell line (KPC-P) and the control KPC line (KPC), but not in the IL-6 KO line (KPC IL6^KO^; Fig. 2 A); loss of expression was due to an insertional mutation (Fig. S2 B). Both control and KO lines exhibited similar growth characteristics in vitro (Fig. 2 B), indicating that autocrine IL-6 was not required for proliferation.

We modeled cachexia in vitro by application of KPC conditioned media (CM) to myotubes differentiated from C2C12 myoblasts (Fig. 2 C). Previously, we showed that IL-6 induces atrophy of C2C12 myotubes via a STAT3-dependent mechanism (Bonetto et al., 2012). Here, CM from KPC cells induced STAT3 phosphorylation (Fig. 2 D) and reduced myotube diameter by 25% (Fig. 2, E and F). In contrast, KPC-IL6^KO^ CM failed to induce pSTAT3 or reduce myotube diameter. IL-6 neutralizing antibody (2 mg/ml) blocked KPC CM-induced myotube wasting, while addition of IL-6 (300 pg/ml) to KPC IL6^KO^ CM restored myotube atrophy (Fig. 2, G and H), an activity that was also blocked by IL-6 neutralizing antibody (Fig. 2 H). Thus IL-6 in KPC cells is responsible for the wasting activity on myotubes.

KPC and KPC IL6^KO^ cells were implanted in the pancreases of male C57BL6/J mice. IHC demonstrated IL-6 expression in stromal compartments in both tumor types. However, only KPC cells (Fig. 2 I, left) were positive for IL-6, while KPC IL6^KO^ tumor cells showed no immunoreactivity (Fig. 2 I, right). Deletion of tumor cell–derived IL-6 increased mouse survival, with median survival of 24 d in KPC IL6^KO^ tumor mice compared with 17 d in KPC tumor mice (P = 0.001; Fig. 2 J).

### Muscle wasting is spared in KPC IL6^KO^ tumor–bearing mice

**Figure 3.**
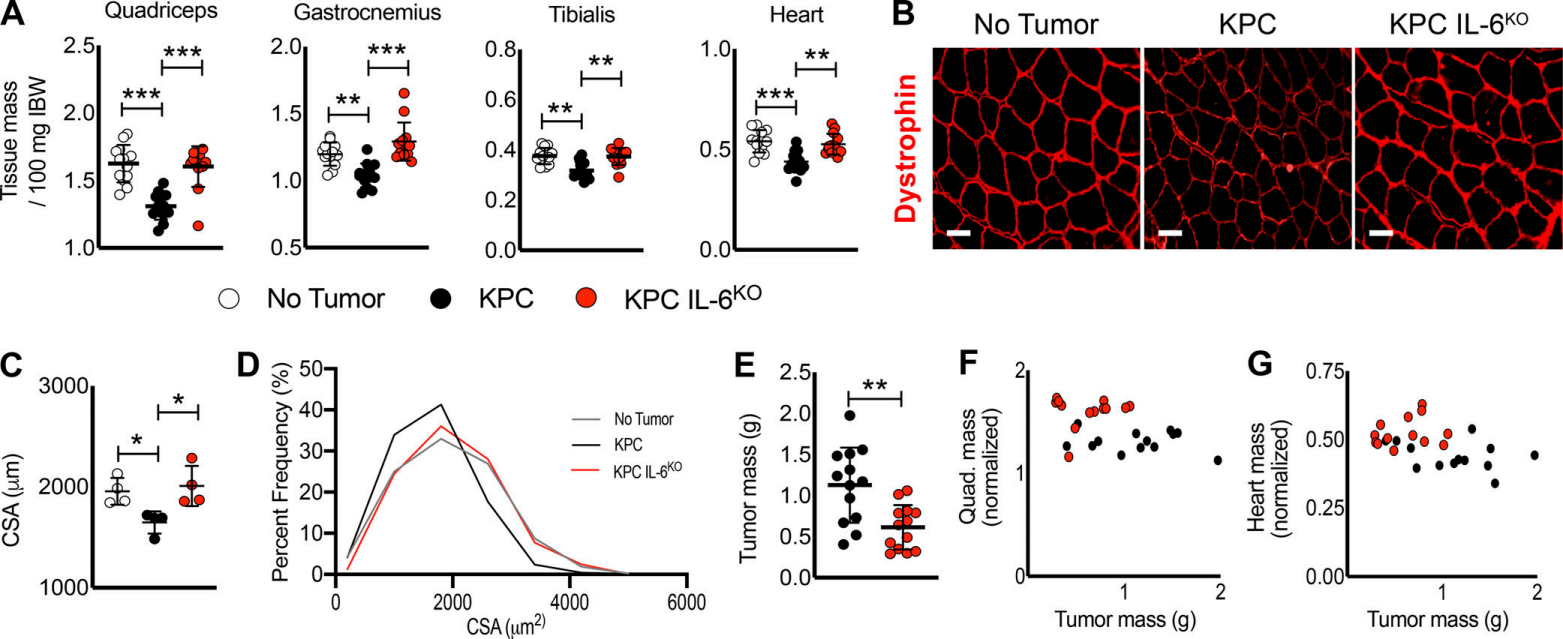
**Deletion of tumor cell IL-6 attenuates muscle wasting.** **(A)** KPC tumor–bearing mice reached our humane endpoint criteria 17 d after injection, and all groups were euthanized simultaneously. Skeletal muscles and the heart were excised at euthanasia and weighed and normalized to initial body weight (IBW). Data represent the mean plus SD from two individual experiments (*n* = 13 per group); significant differences (**, P < 0.005; ***, P < 0.0005) were determined using ANOVA and Tukey’s multiple comparisons test. **(B and C)** Evaluation of muscle fiber CSA was done using sections from excised quadriceps muscles from individual mice reacted for dystrophin expression (B), and mean fiber CSA was determined by comparing myofiber means from muscle cross sections from a subset of samples (*n* = 4; *n* > 300 myofibers per animal), quantified from 20 random fields from each cross section (C). Scale bar = 50 µm. Error bars are mean and SD, and significant differences (*, P < 0.05) were determined using ANOVA and Tukey’s multiple comparisons test. **(D)** Cumulative fiber CSA values from mice in each group were organized based on percent distribution of fiber CSA and plotted to observe shifts in distribution. **(E)** Tumors were excised, and tumor mass was recorded. Data represent the mean plus SD from two individual experiments (*n* = 13 per group); significant differences (**, P < 0.005) were determined using Student’s *t* test. **(F and G)** Correlation analysis of tumor size and muscle size for the quadriceps and heart showed no correlations.

**Figure S3.**
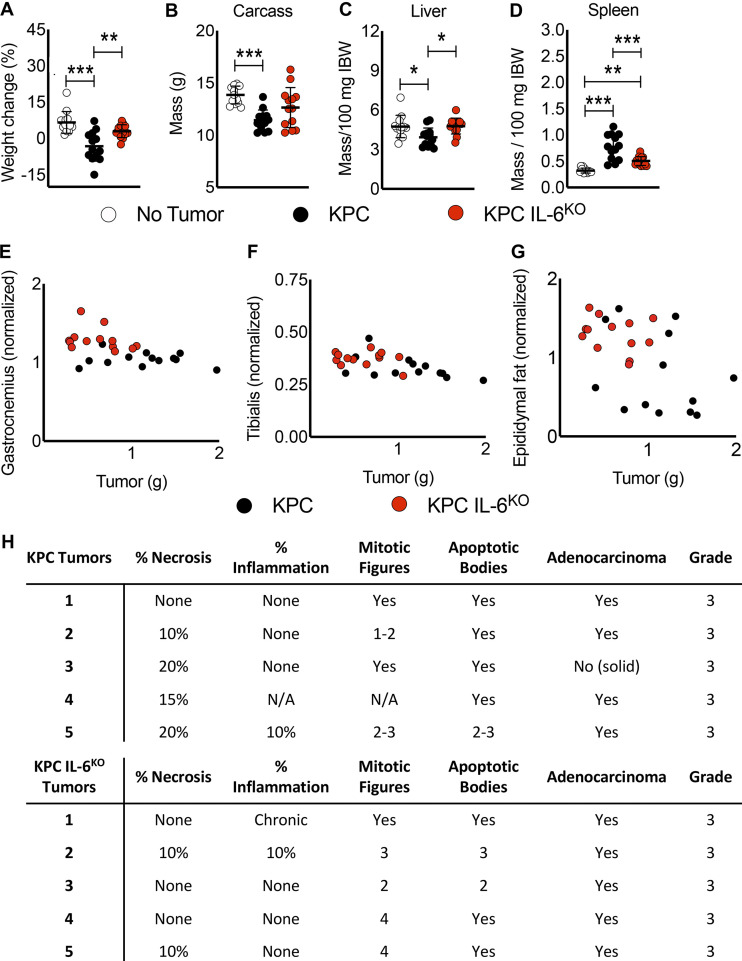
**Deletion of tumor cell–derived IL-6 is associated with reduced weight loss, carcass and liver wasting, and splenomegaly in mice. (A–D)** Tumor-free final body weight was measured at euthanasia (A), and tissue weights for the carcass (B), liver (C), and spleen (D) were also recorded. **(E–G) **No correlation was observed between tumor mass and gastrocnemius (E), tibialis (F), and epididymal fat pad (G) masses after analysis using the Pearson *R* coefficient. Data are from two individual experiments (*n* = 13 per group). **(A–D)** Data represent the mean and SD, and statistical differences (*, P < 0.05; **, P < 0.01; ***, P < 0.001) were determined using ANOVA and Tukey’s multiple comparisons test. **(H)** Sections of excised KPC (*n* = 5) and KPC IL6^KO^ (*n* = 5) tumors were stained with H&E and blindly scored by two separate pathologists to determine any differences in tumor grade; N/A indicates no observation. IBW, initial body weight.

In addition to the survival study, two additional trials of matched experimental cohorts were analyzed for effects on cachexia, euthanizing all mice when one group reached specified humane endpoints. Cachexia was prominent in the KPC group, with 11–26% wasting of limb muscles and heart (Fig. 3 A), with significant loss of body weight (Fig. S3 A) and loss of carcass mass (Fig. S3 B). This degree of wasting would be considered severe cachexia. In contrast, muscle weights in the KPC IL6^KO^ group were unchanged versus tumor-free control mice (Fig. 3 A), with no change in body weight or carcass mass (Fig. S3, A and B). KPC tumor–bearing mice also demonstrated liver wasting (Fig. S3 C) and splenomegaly versus sham and KPC IL6^KO^ tumor–bearing mice (Fig. S3 D). KPC IL6^KO^ tumor–bearing mice exhibited no liver wasting and reduced splenomegaly relative to KPC mice (Fig. S3 D).

Morphometric analysis of quadriceps sections showed mice with KPC IL6^KO^ tumors were spared the myofiber atrophy observed in those with KPC tumors (Fig. 3, B and C). Immunofluorescence (IF) staining for dystrophin in quadriceps muscles showed reduced signal in muscle of mice with KPC tumors, as described for mice with other forms of cancer cachexia (Fig. 3 D; Acharyya et al., 2005; Stephens et al., 2015). In contrast, muscle from mice with KPC IL6^KO^ tumors showed normal dystrophin expression (Fig. 3 D).

Mice bearing the KPC tumor had larger tumors than mice with KPC IL6^KO^ tumors (Fig. 3 E). Because cachexia sometimes scales with tumor burden in mouse models, we compared tumor mass with normalized muscle mass. Here, there were no correlations between tumor and quadriceps mass (Fig. 3 F) or tumor and heart mass (Fig. 3 G). Neither were correlations of tumor size with other muscle groups (Fig. S3, E and F) or epididymal fat pad mass (Fig. S3 G) observed. Blind scoring by pathologists in two independent evaluations demonstrated no difference in tumor histology and grade between KPC and KPC IL6^KO^ tumors (Fig. S3 H). Collectively, these results are consistent with IL-6 as a KPC-derived mediator of cachexia and mortality.

### Tumor cell deletion of IL-6 reduces muscle atrophy pathway activation

**Figure 4.**
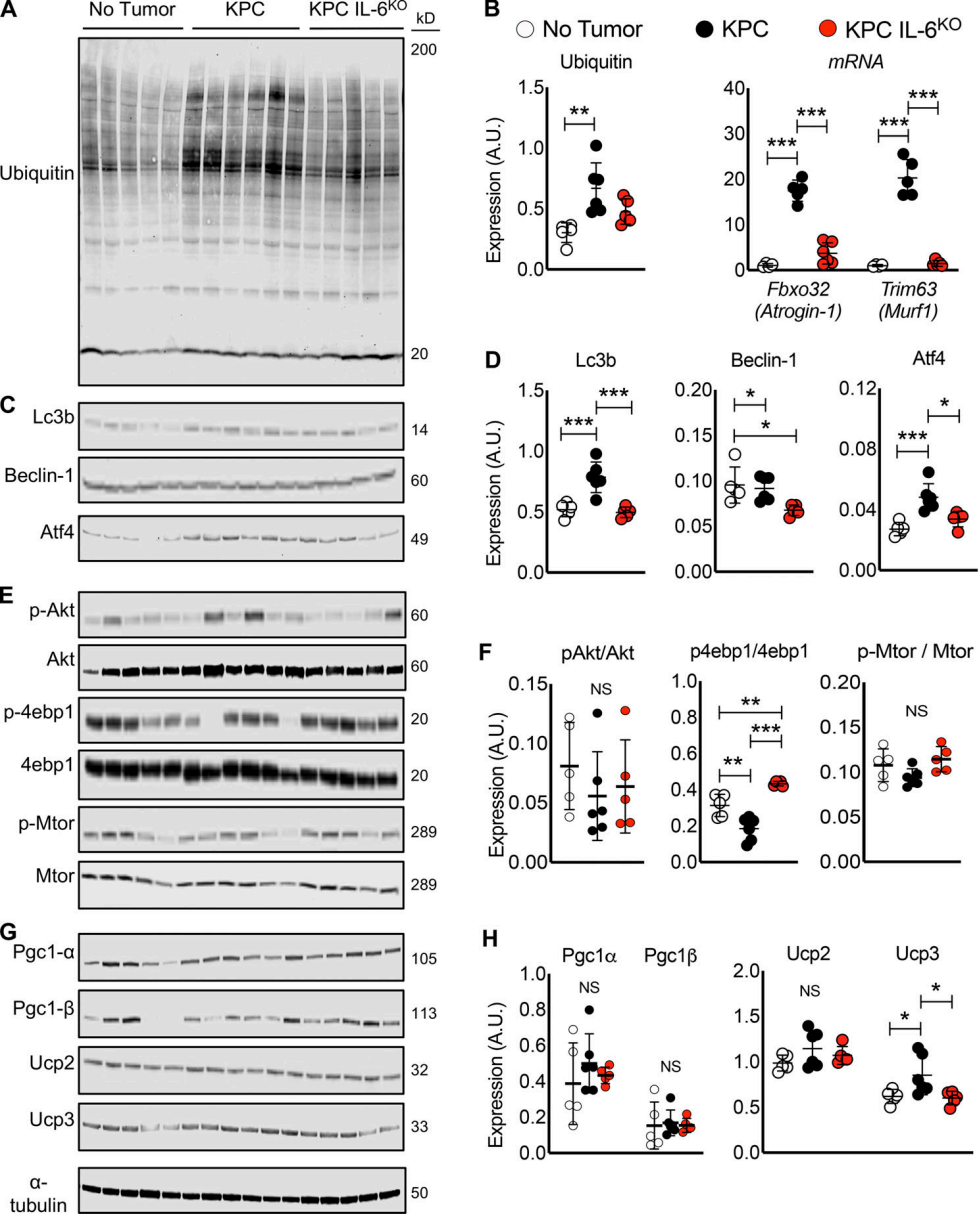
**Measurement of protein expression for common molecular pathways associated with muscle wasting.** **(A–H)** Western blotting results evaluating muscle proteins involved in ubiquitination (A and B, left), autophagy (C and D), anabolism (E and F), and mitochondria biogenesis and metabolism (G and H) from protein lysates made from quadriceps harvested at euthanasia. Results are from a single experiment with no tumor (*n* = 5), KPC (*n* = 6), and KPC IL6^KO^ (*n* = 5) tumor mice, and statistical differences (*, P < 0.05; **, P < 0.01; ***, P < 0.001) were determined using ANOVA and Tukey’s multiple comparisons test. Analysis of qPCR results for mRNA expression of E3 ubiquitin ligases *Atrogin-1* and *Murf1* in quadriceps was also performed (B, right). Data are from a single experiment with *n* = 5 per group, and statistical differences (***, P < 0.0001) were determined with ANOVA and Tukey’s multiple comparisons test. Error bars are mean and SD.

Muscle of mice with KPC IL6^KO^ tumors also showed reduced activation of pro-atrophic pathways that are typically activated in cancer cachexia, including those involved in ubiquitin-proteasome–mediated proteolysis, autophagy, translation repression, and mitochondrial dysfunction. Quadriceps from mice with KPC tumors demonstrated increased total protein ubiquitination, as commonly observed in conditions of muscle catabolism, while total protein ubiquitination in mice with KPC IL6^KO^ tumors was no different from controls (Figs. 4, A and B, left). Consistent with the ubiquitination results, RNA levels for the atrophy-associated skeletal muscle E3 ubiquitin ligases *Fbxo32/Atrogin-1/MAFbx* and *Trim63/Murf1* were only increased in KPC and not in KPC IL6^KO^ muscles (Fig. 4 B, right). Autophagy and ER stress are also triggered in muscle atrophy marked by LC3B-II and Beclin-1, proteins with important roles in autophagosome formation (Sandri, 2016), and Atf4, which is increased by ER stress and the unfolded protein response (Bohnert et al., 2016). LC3B-II and ATF4 protein were increased in the muscle of KPC but not KPC IL6^KO^ tumor–bearing mice (Fig. 4, C and D), while Beclin-1 was unchanged in muscle of mice with KPC tumors and actually decreased in muscle of mice with KPC IL6^KO^ tumors (Fig. 4, C and D). Effects of these KPC tumors on protein synthesis markers in quadriceps were subtle, with no significant change in anabolic phosphorylated protein kinase B (pAKT)/AKT and phosphorylated mechanistic target of rapamycin (p-mTOR)/mTOR (Fig. 4, E and F), and significant decrease in p-4EBP1 in KPC muscle was suggestive of de-repression of translation (Fig. 4, E and F). There was no difference across groups in the master regulators of mitochondrial biogenesis, PGC1A and PGC1B (Fig. 4, G and H). While there was no difference in the mitochondrial uncoupling protein UCP2, an increase in UCP3 was observed in mice with KPC tumors but not in those with KPC IL6^KO^ tumors (Fig. 4, G and H). These results indicate that deletion of IL-6 from tumor cells largely abolished activation of muscle wasting pathways, especially those involved in the ubiquitin-proteasome and autophagy.

### KPC but not KPC IL6^KO^ tumors induced inflammation, lipid accumulation, and oxidative stress in skeletal muscle

**Figure 5.**
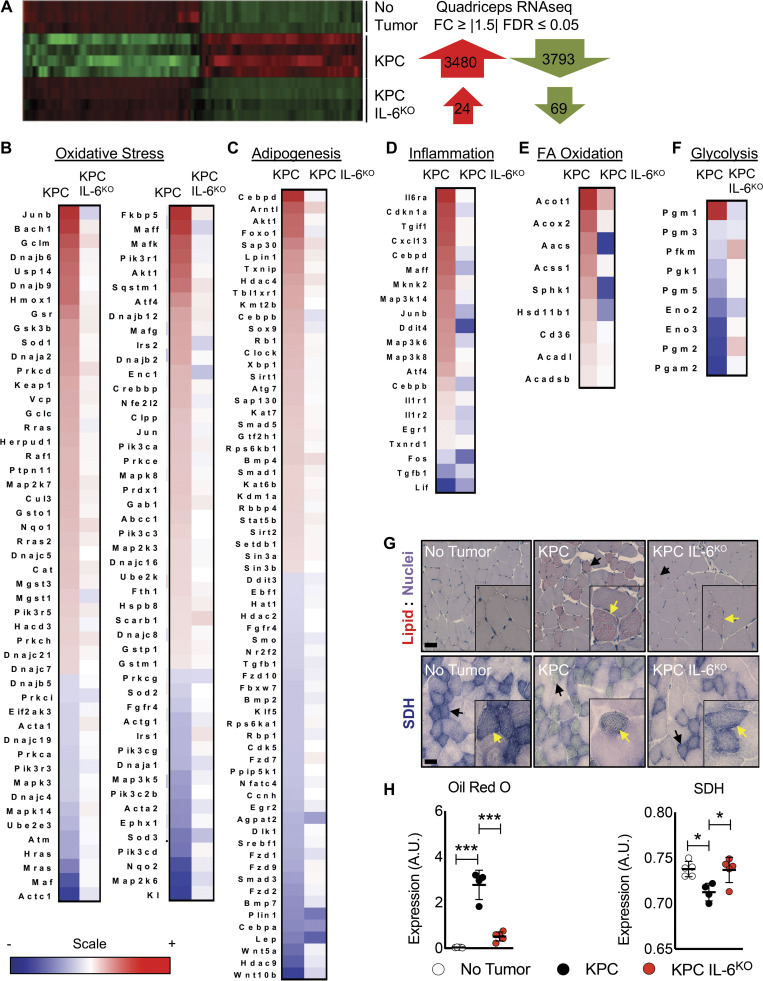
**Deletion of IL-6 from KPC cells reduces activation of key cachexia pathways in muscle.** **(A)** Isolated RNA from the quadriceps of no tumor (*n* = 3), KPC tumor (*n* = 4), and KPC IL6^KO^ tumor (*n* = 4) mice was sequenced, and differentially regulated genes (FC ≥ |1.5| and FDR ≤ 0.05) were compared across groups. **(B–F)** Ingenuity Pathway Analysis using quadriceps RNA-seq data identified various altered pathways and their associated genes (shown in heat map format) that have roles in muscle wasting, including oxidative stress (B), adipogenesis (C), inflammation (D), FA oxidation (E), and glycolysis (F). The scale bar illustrates increased (red) and decreased (blue) gene expression for the heat maps. **(G and H)** Measurements of muscle lipids using ORO staining (G, top, and H, left) and SDH activity as a marker for mitochondria oxidative capacity (G, bottom, and H, right) were performed on quadriceps muscle cross sections (*n* = 5 per group). Scale bar = 50 µm; black arrows in G indicate fibers with increased lipid accumulation and aberrant SDH reactivity at lower magnification; yellow arrows in G indicate fibers with increased lipid accumulation and aberrant SDH reactivity in higher magnification insets. Error bars represent mean and SD, and significant differences (*, P < 0.05; ***, P < 0.0001) were determined using ANOVA and Tukey’s multiple comparisons test.

Muscles of KPC tumor–bearing mice revealed 3,480 up-regulated and 3,793 down-regulated genes by RNA sequencing (RNA-seq) versus shams (fold-change [FC] ≥ ∣1.5∣; false discovery rate [FDR] ≤ 0.05), while the muscles of mice with KPC IL6^KO^ tumors were largely normal, with only 24 up-regulated and 69 down-regulated genes versus shams (Fig. 5 A). Pathway analysis implicated oxidative stress, adipogenesis, inflammation, fatty acid (FA) oxidation (all generally increased in KPC muscle), and glycolysis, which was decreased (Fig. 5, B–F). These were largely unchanged in mice with KPC IL6^KO^ tumors, save FA oxidation, which was decreased relative to controls (Fig. 5 E). Consistent with the pathway analysis, Oil Red O (ORO) staining demonstrated increased intramyocellular lipid accumulation (myosteatosis) in quadriceps of KPC tumor–bearing mice, but not in KPC IL6^KO^ tumor–bearing versus control mice (Fig. 5, G and H, top and left, respectively). Succinate dehydrogenase (SDH) histochemistry, an indicator of mitochondrial respiration and localization, revealed aberrant localization and significantly decreased respiratory capacity in the muscle of KPC tumor mice compared with muscle from KPC IL6^KO^ mice and shams (Fig. 5, G and H, bottom and right, respectively).

### Adipose tissue is not preserved by tumor cell deletion of IL-6

**Figure 6.**
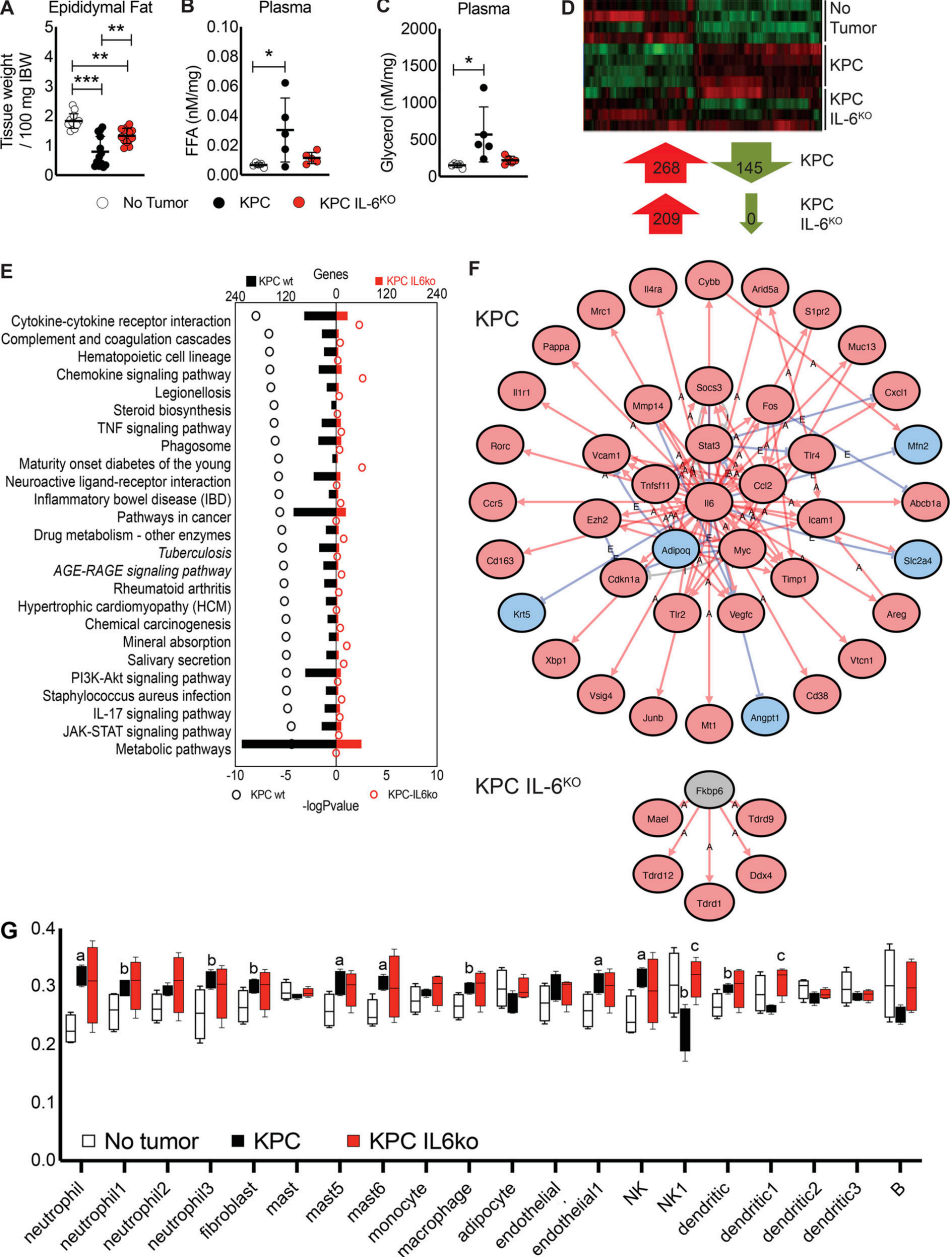
**Deletion of IL-6 from KPC cells reduces fat wasting and alters affected molecular pathways and upstream regulators in adipose tissue.** **(A)** Epididymal fat pad mass was measured at euthanasia and normalized to initial body weight (IBW). Data represent the mean and SD from two separate experiments (*n* = 13 per group), and statistical differences (**, P < 0.01; ***, P < 0.0001) were determined using ANOVA and Tukey’s multiple comparisons test. FFA, free fatty acid. **(B and C)** Characterization of lipolysis using measurements of plasma glycerol (B) and FAs (C) normalized to epididymal fat pad mass from mice. Data represent mean and SD from a single experiment (*n* = 5 per group), and statistical differences (*, P < 0.05) were determined using ANOVA and Tukey’s multiple comparisons test. **(D)** Isolated RNA from the epididymal fat pads (*n* = 4 per group) was sequenced, and differentially regulated genes (compared with no tumor group, FC ≥ |1.5| and FDR ≤ 0.05) were compared across groups. **(E)** iPathway analysis using epididymal fat pad RNA-seq data identified differentially regulated pathways. **(F)** iPathway also identified upstream regulators in the adipose tissue of KPC and KPC IL6^KO^ tumor mice. Red arrows labeled with A indicate an activating interaction, while blue arrows labeled with E indicate an inhibitory interaction. **(G)** Deconvolution analysis of the RNA-seq data identified changes in cell subtypes present in the adipose tissue; mast, mast cells; B, B cells; a, P < 0.05 versus no tumor; b, P < 0.10 versus no tumor; and c, P < 0.05 versus KPC.

**Figure S4.**
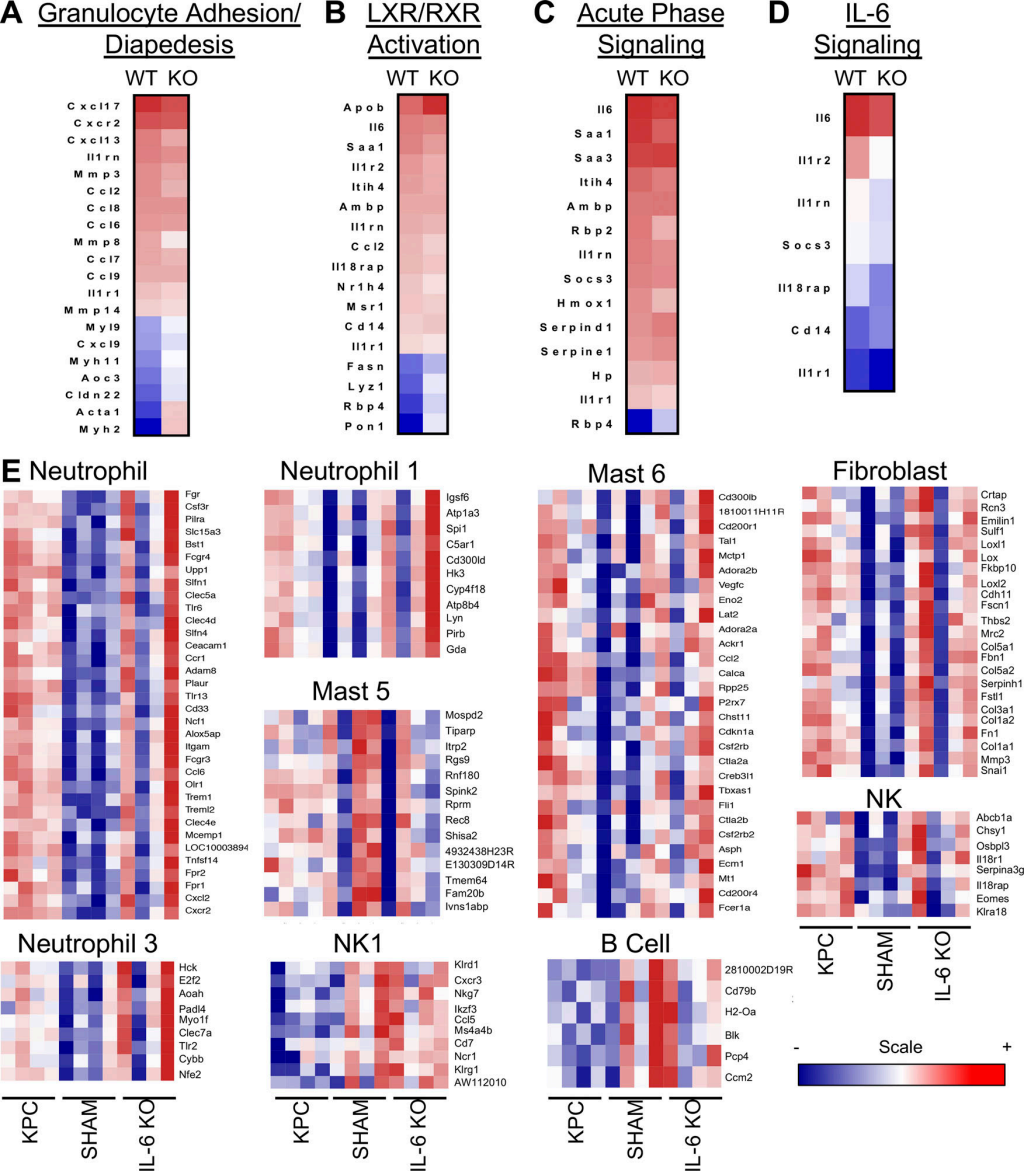
**Characterization of changes in signaling pathways and immune cell populations using RNA-seq from adipose tissue in tumor-bearing mice. (A–D)** Analysis of RNA-seq data from adipose tissue using****Ingenuity Pathway Analysis, which identified various altered pathways and their associated genes (shown in heat map format) that have roles in inflammation and lipolysis, including granulocyte adhesion/diapedesis (A), liver X receptor/retinoid X receptor (LXR/RXR) activation (B), acute phase signaling (C), and IL-6 signaling (D). **(E)** A list of immune cell subtypes and their identifying gene sets that are differentially regulated in adipose tissue in the presence of PDAC as determined using deconvolution analysis of adipose RNA-seq data. The scale bar illustrates increased (red) and decreased (blue) gene expression compared with no tumor mice for the heat maps. Data represent single experiments.

Because IL-6 has been shown to promote lipolysis, we evaluated tumor effects on adipose tissue wasting. Both KPC and KPC IL6^KO^ tumor mice showed wasting of the epididymal fat pad versus shams, although fat wasting in KPC IL6^KO^ tumor mice was significantly attenuated (Fig. 6 A). Consistent with fat wasting, plasma FAs and glycerol were increased, but only in the KPC tumor mice (Fig. 6, B and C). RNA-seq of adipose tissue demonstrated similar transcriptomes between tumor groups, with 268 up-regulated and 145 down-regulated genes in KPC mice and 209 up-regulated and zero down-regulated genes in the KPC IL6^KO^ group (FC ≥ ∣1.5∣; FDR ≤ 0.05; Fig. 6 D). Ingenuity pathway analysis revealed granulocyte adhesion/diapedesis, liver X receptor/retinoid X receptor activation, acute phase signaling, and IL-6 signaling among the top pathways associated with cachexia in adipose tissue (Fig. S4, A–D), with changes in gene expression similar across both tumor conditions, while iPathway highlighted cytokine signaling, metabolism, and JAK-STAT signaling (Fig. 6 E). These same pathways were largely unchanged in the fat of KPC IL6^KO^ tumor mice. Upstream regulator prediction revealed IL-6 identified as the top upstream regulator in the fat of KPC tumor mice (Fig. 6 F); neither IL-6 nor STAT3 was discovered among upstream regulators in adipose of mice with KPC IL6^KO^ tumors. Computational deconvolution of the bulk RNA-seq identified differences in neutrophil, natural killer (NK), endothelial, and mast cell populations and trending differences in fibroblasts, macrophages, and dendritic cells in KPC adipose versus shams (Fig. 6 G; Lu et al., 2020). KPC IL6^KO^ adipose showed no differences from no tumor adipose, but significant differences from KPC in NK1 and dendritic cells. The full comparison as well as specific gene lists used to classify each cell subtype and expression levels are shown (Fig. S4 E). These results indicate that adipose tissue is more sensitive than skeletal muscle to tumor effects, including plasma IL-6 levels, and that adipose tissue exhibits significant infiltration of inflammatory immune cells in PDAC cachexia, which is reduced in the absence of tumor-derived IL-6.

### IL-6 pathway proteins are differentially expressed in fat versus muscle of mice with PDAC, implicating IL6R trans-signaling from muscle to fat

**Figure 7.**
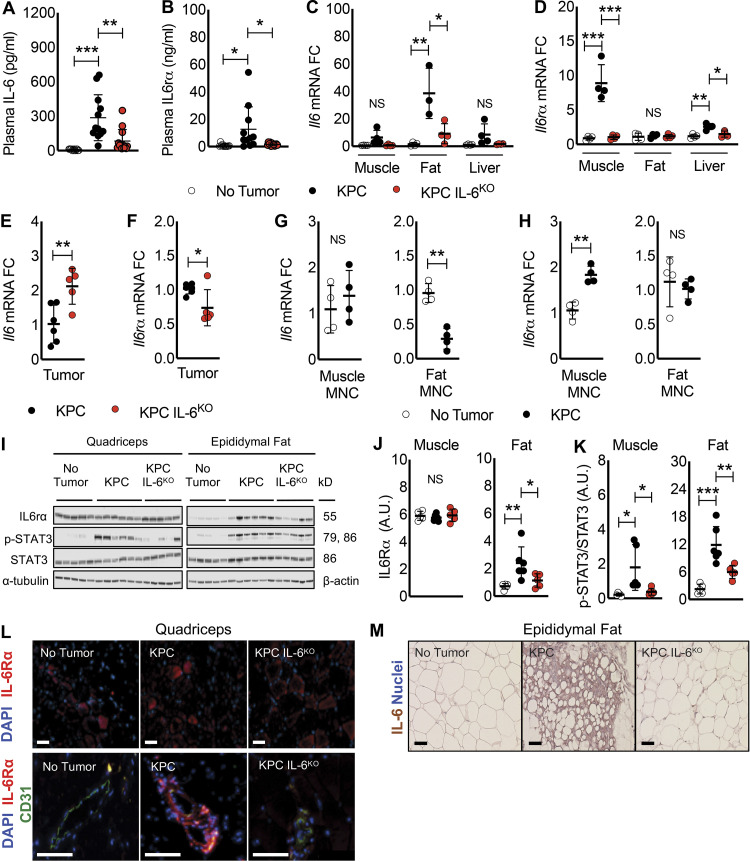
**Evidence for an IL-6, IL6R circuit among tumor, adipose tissue, and skeletal muscle in PDAC cachexia.** **(A and B)** Plasma from no tumor, KPC tumor, and KPC IL6^KO^ tumor mice was harvested immediately before euthanasia and measured for IL-6 (A) and IL6R (B) protein expression using an ELISA. Data represent mean and SD from two separate experiments (*n* = 13 per group), and statistical differences (*, P < 0.05; **, P < 0.001; ***, P < 0.0001) were determined using ANOVA and Tukey’s multiple comparisons test. **(C and D)** Isolated RNA from quadriceps, epididymal fat, and liver of mice was used to measure mRNA expression of *Il6* (C) and *Il6r* (D) in tissues and are presented as FC versus no tumor mice. Data represent mean and SD from a single experiment, and statistical differences (*, P < 0.05; **, P < 0.01; ***, P < 0.001) were determined using ANOVA and Tukey’s multiple comparisons test. **(E and F)** Isolated RNA from KPC (*n* = 6) and KPC IL6^KO^ (*n* = 5) tumors was used to measure mRNA expression of *Il6* (E) and *Il6r* (F) in tumors and are presented as FC versus KPC tumors. Data represent mean and SD from a single experiment, and statistical differences (*, P < 0.05; **, P < 0.01) were determined using ANOVA and Tukey’s multiple comparisons test. **(G and H)** To determine with increased detail the source of *Il6 *and *Il6r* mRNA in muscle and fat, mononuclear (MNC) cell fractions were dissociated from whole tissue, and isolated RNA from the MNC fraction was measured for *Il6* (G) and *Il6r *(H) mRNA expression. Data represent mean and SD from a single experiment with *n* = 4 per group, and statistical differences (**, P < 0.01) were determined using Student’s *t* test. **(I–K)** Protein expression for IL6R and STAT3 phosphorylation (p-STAT3) was quantified in the quadriceps and epididymal fat pads of no tumor (*n* = 5), KPC (*n* = 6), and KPC IL6^KO^ (*n* = 5) mice using Western blotting. Data represent the mean and SD for a single experiment, and statistical differences (*, P < 0.05; **, P < 0.01; ***, P < 0.001) were determined using ANOVA and Tukey’s multiple comparisons test. **(L)** IF showing the expression of IL6R protein (red) and nuclei (blue, DAPI) in the quadriceps muscle fibers of mice (L, top; scale bar = 50 µm) and IL6R protein (red), the endothelial protein marker CD31 (green), and nuclei (blue, DAPI) to visualize IL6R expression in and around blood vessels in the quadriceps muscle of mice (L, bottom**;** scale bar = 100 µm). **(M)** IHC was used to visualize IL-6 protein localization in the epididymal fat pads. Scale bar = 50 µm.

Given the differential sensitivity of muscle and fat to the presence of tumor-derived IL-6, we sought to investigate tissue crosstalk in PDAC cachexia. Plasma levels of IL-6 were significantly increased (∼150-fold) in KPC tumor mice over normal control levels; while mice with KPC IL6^KO^ tumors showed increased plasma IL-6, the difference was not significantly different from shams (Fig. 7 A). Plasma sIL6R, which is largely produced by shedding of a 55-kD fragment from the membrane-bound IL6R, was also significantly increased in KPC tumor mice (∼25-fold) over shams, while plasma sIL6R levels in KPC IL6^KO^ mice were not different from controls (Fig. 7 B).

**Figure S5.**
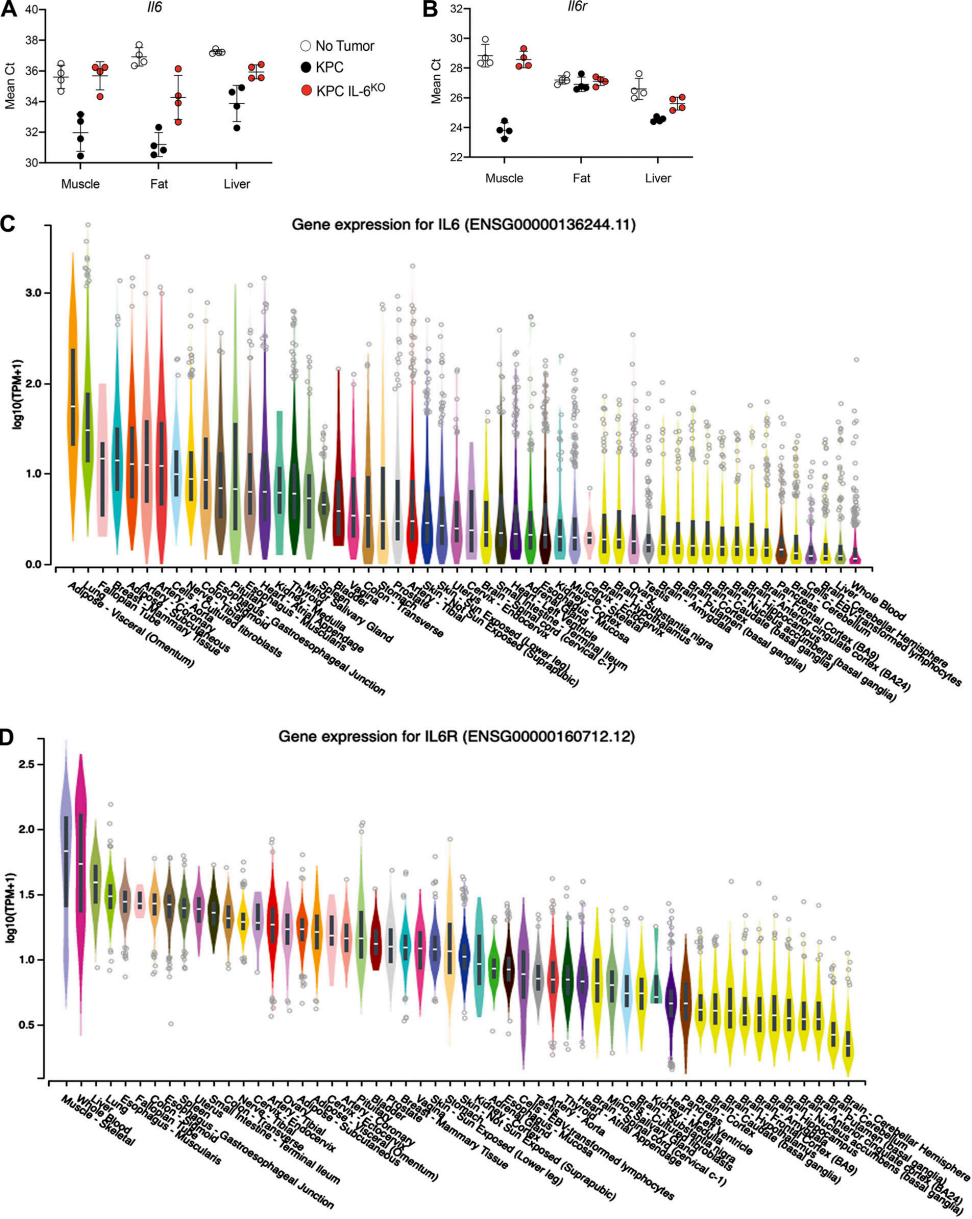
**Tissue Il6 and Il6r mRNA expression using mean Ct from qPCR and the GTEx Portal database support fat as a significant source of Il6 and muscle as a significant source of Il6r. (A) **Comparison of *Il6 *mean Ct from qPCR analysis between groups for muscle, fat, and liver shows fat from KPC tumor mice with the lowest mean Ct, indicating the highest expression of *Il6* among tissues analyzed. **(B) **Comparison of *Il6r *mean Ct from qPCR analysis between groups for muscle, fat, and liver shows muscle from KPC tumor mice with the lowest mean Ct, indicating the highest expression of *Il6r* among tissues analyzed. In A and B, error bars are mean plus SD. **(C)** Data represent single experiments. Human *Il6 *mRNA expression across various tissues according to the GTEx Portal Database shows adipose tissues as having the highest *Il6 *expression.** (D)** Human *Il6r *mRNA expression across various tissues according to the GTEx Portal Database shows skeletal muscle as having the highest *Il6r *expression.

To identify the sources of IL-6 and sIL6R, we measured *Il6 *and *Il6r *mRNA expression in muscle, fat, liver, and tumor. In mice with KPC tumors, *Il6* mRNA expression was elevated in skeletal muscle, though not meeting our a priori definition of significance (6.4-fold, P = 0.06), and liver (8.5-fold, P = 0.1) and was significantly increased (37-fold) in epididymal fat (Fig. 7 C). In contrast, *Il6r *mRNA was significantly increased in the quadriceps (8.9-fold, P = 0.0001) and liver (2.6-fold, P = 0.002) but was unchanged in the adipose tissue from KPC tumor mice (Fig. 7 D). Compared with KPC tumors, *Il6 *mRNA was increased in the KPC IL6^KO^ tumors (Fig. 7 E), presumably due to enhanced expression of *Il6 *in stroma (see Fig. 2 I, IHC), while *Il6r* mRNA was decreased in KPC IL6^KO^ versus KPC tumors (Fig. 7 F). To further verify that *Il6 *was predominantly induced in fat and *Il6r* predominantly induced in skeletal muscle, mean cycle threshold (Ct) was plotted for each gene across tissues. Indeed, fat from KPC tumor mice had the lowest mean Ct for *Il6*, and skeletal muscle from KPC tumor mice had the lowest mean Ct for *Il6r* (Fig. S5, A and B), implicating fat and muscle as the predominant sources of *Il6 *and *Il6r *in our model, respectively. Furthermore, the GTEx Portal of human tissue transcriptomes reveals adipose tissue among the highest *Il6-*expressing tissues (Fig. S5 C) and skeletal muscle as the top tissue for *Il6r* (Fig. S5 D), further implicating fat and skeletal muscle as significant sources of IL-6 and IL6R, respectively. We attribute the majority of *Il6 *induction to adipocytes in fat and the majority of *Il6r *to myofibers in muscle and not from nonparenchymal cell types in either tissue because mononuclear cells isolated from those tissues do not demonstrate equivalent induction versus the whole tissue in mice with cachexia versus shams (Fig. 7, G and H; myofibers and adipocytes are eliminated in these mononuclear cell preparations due to size). These results indicate that the mononuclear cells from muscle and fat do not contribute significantly to *Il6 *mRNA and that muscle mononuclear cells contribute a fraction of skeletal muscle *Il6r *mRNA induction in cachexia.

In contrast to mRNA induction in muscle, there was no change in IL6R protein in the quadriceps; moreover, despite no mRNA induction, a significant increase of a 55-kD IL6R protein was observed in the adipose tissue of KPC tumor mice (Fig. 7, I and J). Thus, we infer that *Il6r *mRNA induction results in the production of soluble receptor in muscle (and potentially other tissues) and further that soluble receptor subsequently accumulates in fat. Using pY705-STAT3 as readout of IL-6 signaling, KPC mice showed increased pY705-STAT3 in muscle and fat, while KPC IL6^KO^ tumor–bearing mice showed only increased pY705-STAT3 in fat (Fig. 7, I and K). IF demonstrated IL6R protein in myofibers in all conditions (Fig. 7 L, top) as well as robust staining of muscle blood vessels in the KPC tumor mice (Fig. 7 L, bottom). Strong IL-6 expression was observed in fat from KPC tumor–bearing mice (Fig. 7 M). Taken together, these results implicate fat as a primary source of IL-6 and muscle as a primary source for circulating sIL6R, which ultimately likely accumulates in fat.

### Tissue wasting and *Il6* and *Il6r* mRNA expression are modeled in in vitro studies of tumor–adipose–muscle crosstalk

**Figure 8.**
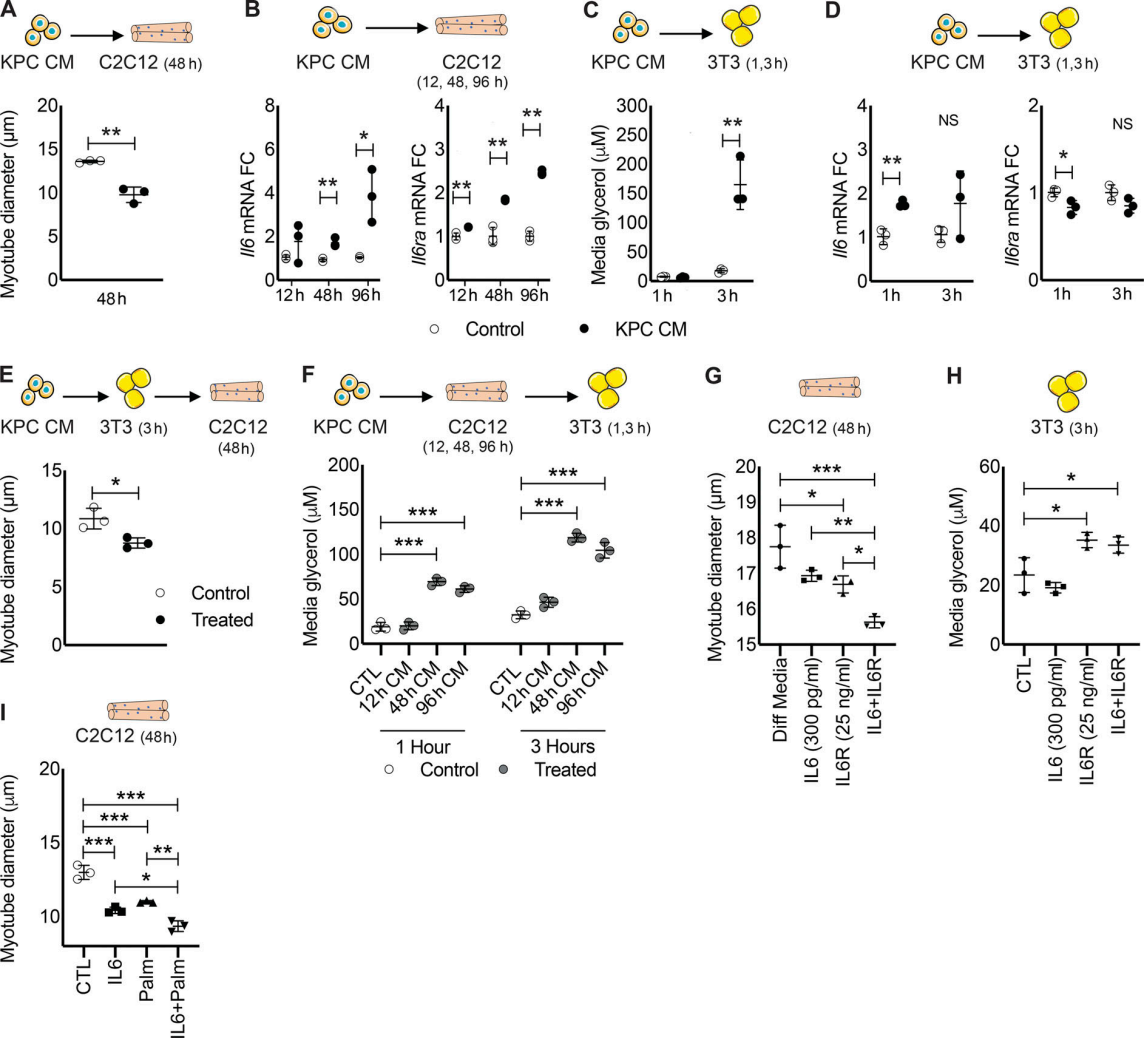
**Modeling of IL-6, IL6R tumor–tissue crosstalk in vitro.** **(A)** C2C12 myotubes were incubated with DM (Control) or CM from KPC tumor cells in triplicate for 48 h, and myotube diameter was measured. **(B)** Myotubes were incubated in triplicate with Control or KPC CM for 12, 24, and 96 h, and isolated RNA was used to measure mRNA expression of *Il6 *and *Il6r* at each time point. **(C)** 3T3 adipocytes were incubated in triplicate with KPC CM for 1 and 3 h, and media glycerol concentration was measured as a marker of lipolysis. **(D)** 3T3 adipocyte RNA was harvested at 1 and 3 h after treatment in triplicate with KPC CM and used to measure *Il6* and *Il6r* mRNA expression. **(E)** The tumor–fat–muscle crosstalk was investigated by incubating adipocytes with KPC CM and then using the adipocyte CM to treat myotubes in triplicate for 48 h and measure myotube diameter. IF was used to measure myotube diameter from 20 random fields per well (n ∼150 diameter measurements per well, *n* = 3 wells). **(A–E)** Data represent the mean and SD from single experiments, and statistical differences (*, P < 0.05; **, P < 0.01) were determined using Student’s *t* test between groups at individual time points. **(F)** The tumor–muscle–fat crosstalk was investigated by treating myotubes for 12, 24, and 96 h with KPC CM and then using the myotube CM to treat adipocytes in triplicate for 1 and 3 h to measure lipolysis via media glycerol content. **(G)** To decipher the effects of IL-6 and IL6R in muscle and fat, myotubes and adipocytes were treated in triplicate in vitro with recombinant IL-6 and IL6R. Myotubes were incubated with DM (Diff Media), physiological levels of recombinant mIL-6, physiological levels of recombinant murine IL6R, or the combination of IL-6 and IL6R for 48 h, and myotube diameter was measured. Myotube diameter was measured as described in E. **(H)** Adipocytes were incubated in triplicate with GM (Control [CTL]), physiological levels of recombinant mIL-6, physiological levels of recombinant murine IL6R, or the combination of IL-6 and IL6R for 3 h, and media glycerol was measured. **(I)** Myotubes were incubated with IL-6 (20 ng/ml), palmitate (Palm; 0.5 mM), and the combination for 48 h, and myotube diameter was measured. Myotube diameter was measured as described in E. **(F–I)** Data represent the mean and SD from single experiments, and statistical differences (*, P < 0.05; **, P < 0.01; ***, P < 0.001) were determined using ANOVA and Tukey’s multiple comparisons test.

Our results thus far demonstrate that KPC tumors increase lipid accumulation, *Il6r* expression, and atrophy in skeletal muscle, while also activating lipolysis and *Il6 *expression**in fat. These effects on distant tissues could be mediated directly by tumor-derived products or indirectly through other cellular or molecular mediators. Thus, we tested whether products of KPC cells could affect muscle and fat directly. KPC CM induced myotube atrophy (Fig. 8 A) and, as observed in mice, increased myotube *Il6* and *Il6ra *mRNA expression**(Fig. 8 B). KPC CM also increased lipolysis in 3T3-L1 adipocytes as measured by increased media glycerol concentration (Fig. 8 C). Consistent with our in vivo results, *Il6* but not *Il6ra *mRNA expression was also increased in adipocytes after treatment with KPC CM (Fig. 8 D).

KPC-induced adipose wasting produced increased circulating FAs and glycerol in vivo (Fig. 6, B and C). While extracellular lipids have been implicated in muscle dysfunction in diabetes and metabolic syndrome, these effects in PDAC cachexia are not well described. Thus, we aimed to model tumor-to-fat-to-muscle crosstalk in vitro using CM swapping studies. Adipocytes were treated for 1 h with KPC CM to stimulate lipolysis, then washed and replenished with normal growth medium, which was collected after 3 h and used to treat myotubes for 48 h (Fig. 8 E, top). This KPC-activated adipocyte–CM induced myotube atrophy (Fig. 8 E). To investigate tumor-to-muscle-to-fat crosstalk, myotubes were treated with KPC CM for 12, 48, and 96 h, and the resulting myotube CM was then used to treat adipocytes (Fig. 8 F, top). KPC-activated myotube CM significantly induced lipolysis at 1 and 3 h, as evidenced by increased glycerol in the adipocyte media (Fig. 8 F).

**Figure 9.**
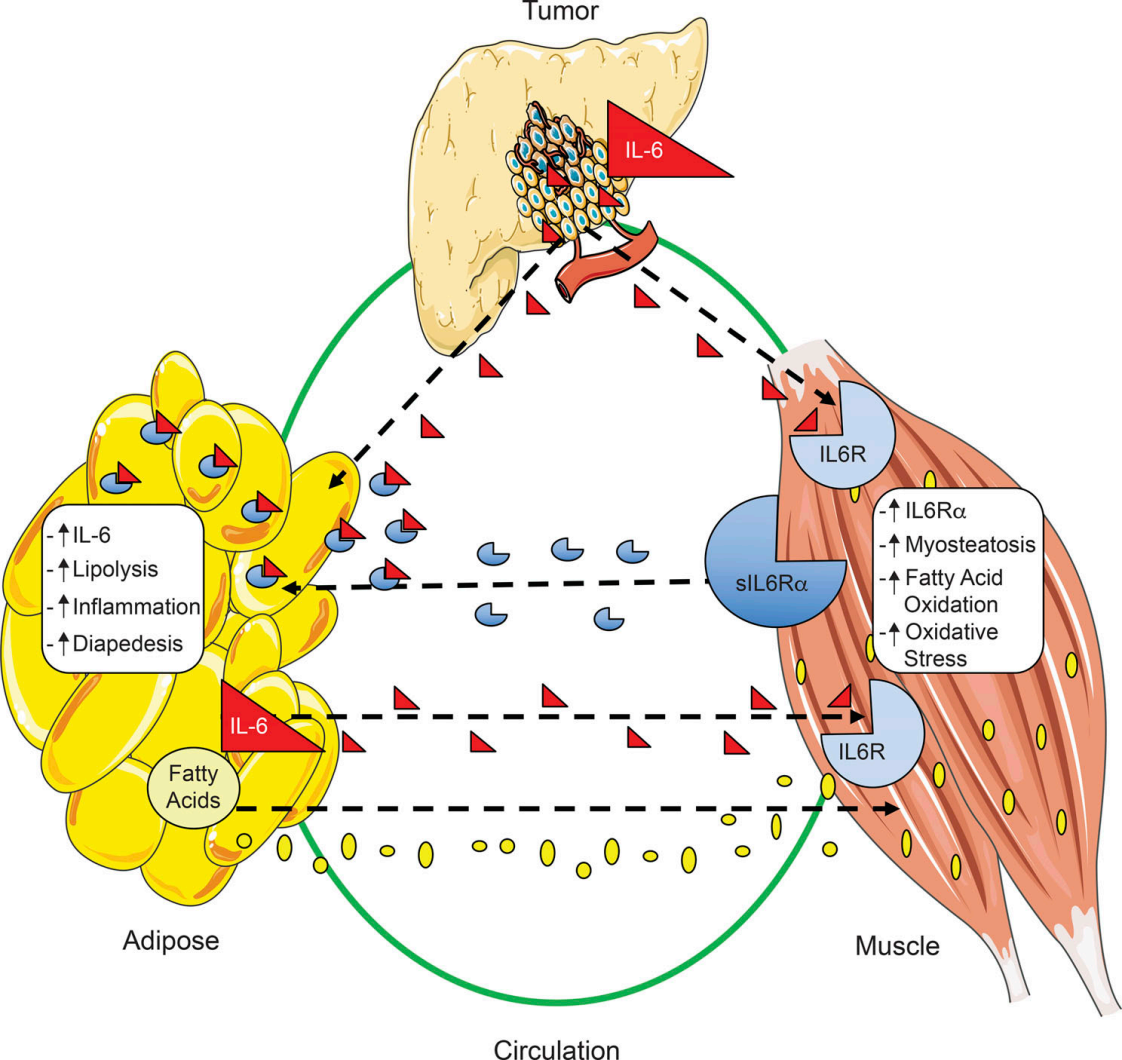
**Model of tumor–fat–muscle crosstalk in PDAC. **Summary of the crosstalk between tumor, muscle, and fat in PDAC-associated cachexia. IL-6 produced by tumor epithelial cells acts to exacerbate both fat and muscle wasting, leading to myosteatosis and systemic inflammation as a result of increased lipolysis and production of IL-6 and FAs by fat. Production of IL6R by muscle is increased in the presence of PDAC and contributes to elevated sIL6R levels in plasma. Ultimately, the increases in both FAs and sIL6R in plasma act in a feed-forward mechanism, where muscle and fat contribute to each other’s wasting in PDAC cachexia.

Next, we investigated whether physiological concentrations of IL-6, sIL6R, or the combination could induce wasting in both myotubes and adipocytes. Myotube wasting was observed in the presence of either IL-6 (300 pg/ml) or sIL6R (25 ng/ml) versus controls; however, the combination of IL-6 and sIL6R produced the most severe atrophy, which was roughly additive versus either alone (Fig. 8 G). In adipocytes, physiological levels of exogenous IL-6 did not induce lipolysis, while addition of sIL6R was sufficient to induce lipolysis with or without added IL-6 (Fig. 8 H). These results indicate that IL6R is limiting for IL-6–mediated lipolysis in adipocytes and that lipolysis is mediated via IL-6 trans-signaling. Finally, we tested the combination of supra-physiological levels of IL-6 and palmitate (to model FA increases in cachexia) on C2C12 myotubes. Each of these factors induced myotube atrophy alone, while the combination was additive (Fig. 8 I). These findings demonstrate that both IL-6 signaling and lipids can mediate muscle loss and, further, that IL-6–mediated activation of adipose lipolysis via trans-signaling likely contributes to muscle wasting in PDAC (Fig. 9).

## Discussion

While the majority of patients with PDAC suffer cachexia, not everyone does. Understanding this heterogeneity of phenotypes at molecular, cellular, and tissue levels could lead to treatments for this currently unmet clinical need (Roeland et al., 2020). Here, we provide evidence of a central role for tumor cell–derived IL-6 in mediating PDAC cachexia, and we demonstrate a novel tumor–tissue crosstalk mediated through IL-6 and sIL6R trans-signaling in the macroenvironment of the host.

We made cachexia avatars using PDXs, tested the centrality of IL-6 by deleting it from tumor cells, and modeled the resulting effects and deduced tissue crosstalk in fat and muscle using cell culture. We show that the heterogeneity of weight loss before surgery observed in patients with the same diagnosis and similar staging was reproduced in mice implanted with their tumors. Thus, differences in cachexia phenotypes can be modeled in mice and derive in part from tumor-specific characteristics. The most aggressive cachexia was associated with human IL-6 in the mice, pointing to a central role for IL-6 derived from malignant cells in this third passage of the patient tumor fragments. Moreover, morbidity in these models was driven not by tumor growth but rather by the extent of cachexia, suggesting that cachexia drives mortality. By deleting IL-6 from murine tumor cells, we revealed a greater sensitivity of adipose tissue to the presence of tumor and confirmed the functional importance of tumor cell IL-6. We further define mechanisms by which tumor cells elicit cachexia by inducing crosstalk between fat and muscle, mediated via a feed-forward, IL-6 trans-signaling loop. Malignant cells signal via IL-6 to muscle and fat, and muscle via sIL6R to fat, and fat via FAs and IL-6 to muscle, which are all targetable mechanisms for treatment of cachexia (Fig. 9).

Blood levels of IL-6 are generally correlated with poor outcomes and mortality in patients with late-stage PDAC (Holmer et al., 2014; Schultz et al., 2013; Palmquist et al., 2020); however, several studies including our own demonstrate no correlation of circulating IL-6 with cachexia severity in patients with PDAC (Talbert et al., 2018; Ramsey et al., 2019). These discrepancies with observations in mouse models, including our own, might relate to the difficulty of detecting the many forms of IL-6 in circulation, both unbound and bound to sIL6R and sGP130, rather than reflecting the absence of IL-6 activity (Chaturvedi et al., 2015). Indeed, a proteomic signature of IL-6 activity correlates to cachexia severity in patients with PDAC (Narasimhan et al., 2020). Alternatively, tissue levels of IL-6 and its signaling components might be both more relevant as biomarkers and more important for initiating wasting. Biomarker studies of adipose and muscle are ongoing in our group, but tissue-specific depletion of these pathways is required to demonstrate mechanism conclusively.

Cell-type expression of IL-6 might also dictate cachexia phenotypes. IL-6 expression by fibroblasts and myeloid cells in the tumor microenvironment has been implicated in PDAC development and tumor growth, progression, metastasis, drug resistance, and immune suppression (Holmer et al., 2014; Mace et al., 2018; Öhlund et al., 2017). Functions of IL-6 production by malignant tumor cells are much less studied. Our data indicate that a substantial fraction of human PDAC tumors exhibit expression of IL-6 in malignant cells and further that IL-6 is produced by many established PDAC tumor cell lines. In our avatar models, mice exhibiting the highest levels of human IL-6 in plasma also had the highest severity of cachexia and mortality, implicating IL-6 arising from metastatic cells. This relationship was independent of tumor size. In culture, autocrine IL-6 production did not influence KPC cell proliferation, although IL-6 null tumors were smaller in mice. Whether reduced tumor growth in vivo was due to the absence of tumor cell IL-6 or to reduced growth substrates (e.g., amino acids, FAs, glycerol) due to reduced cachexia is a complex question under active investigation in our laboratory. Regardless, IL-6 production from malignant cells can orchestrate cachexia, and this tumor characteristic could have diagnostic and therapeutic implications for PDAC.

IL-6 produced by the tumor was amplified distantly via production of IL-6 from adipose and muscle. Fat loss was proportionate to IL-6; both circulating IL-6 and fat wasting were halved by elimination of IL*-*6 from tumor cells. In contrast, muscle loss was abolished by tumor cell depletion of IL-6. Thus, adipose tissue was more sensitive to the effects of PDAC or IL-6 than was skeletal muscle. Alternatively, muscle wasting could be a consequence of adipose wasting. When fat was lost more completely, muscle exhibited lipid accumulation, disordered mitochondria, oxidative injury, and myofiber atrophy. Consistent with lipid-mediated wasting, palmitate treatment of myotubes here was sufficient to cause myotube atrophy. Consistent with a central role for the tumor in orchestrating this organ interplay, KPC cell media activated adipocytes to produce factors that induced myotube wasting and activated myotubes to produce factors that induced adipocyte lipolysis.

PDAC greatly increased *Il6r* mRNA expression in muscle and modestly in the liver, but not in fat. Instead, sIL6R protein accumulated in fat concomitantly with increased STAT3 phosphorylation, lipolysis, and inflammation, suggestive of sIL6R trans-signaling in adipose tissue. Our in vitro experiments support such crosstalk between these tissues. As in mice, KPC cell media activated adipocyte *Il6 *expression and myotube *Il6* and *Il6r *mRNA expression. Physiological levels (those observed in KPC cachexia) of IL6R elicited adipocyte lipolysis, while physiological levels of IL-6 did not, suggesting that IL6R is limiting for signaling in fat and thus must be supplied by trans-signaling. In contrast, the addition of either IL-6 or IL6R induced myotube atrophy, with the combination of IL-6 and IL6R inducing the most atrophy. Furthermore, supraphysiological levels of IL-6, sIL6R, and palmitate as representative of free FAs resulted in additive levels of atrophy. This suggests that muscle wasting mediated by IL-6 is saturable and parallel to that produced by excess lipid uptake and thus these might be separately targetable pathways at the level of skeletal muscle.

In these studies, skeletal muscle was a major source of sIL6R, given both the dramatic induction of expression and the overall mass of muscle, roughly 40% of body weight. Among human tissues, skeletal muscle expresses the highest level of IL6R mRNA among all tissues tested. Speculatively, sIL6R may translocate from skeletal muscle to other tissues via proteolytic cleavage of membrane GP80, which in mice and humans has been shown to be mediated by ADAM10 and ADAM17 proteases (Schumacher et al., 2015). These proteases could be activated by lipid uptake in skeletal muscle. In diabesity, excess circulating lipids are taken up by muscle, leading to myosteatosis, accumulation of diacylglycerol, activation of PKCθ (Szendroedi et al., 2014), and downstream insulin resistance. PKCθ is known to activate ADAM10 and ADAM17 and increase shedding of the sIL6R (Müllberg et al., 1992). These findings suggest a connection between adipose wasting and myosteatosis-induced IL6R trans-signaling in skeletal muscle that must be tested using genetic approaches. Alternatively, sIL6R could result from increased expression of induced splice variants that lack the transmembrane and intracellular domains (Rose-John, 2015).

Our results in mice and cells suggest that fat loss is an earlier and precipitating event for muscle loss in cachexia. Chronic lipolysis of adipose tissue results in increased circulating FAs and subsequent FA uptake and lipid accumulation in muscle, which, after a period of metabolic adaptation, ultimately results in lipotoxicity, including metabolic derangement, cellular stress, and muscle wasting. Our experimental data support this interpretation. Impaired β-oxidation was highlighted as central to muscle wasting in experimental renal cell carcinoma models, and inhibition of FA oxidation improved body weight and muscle mass in mice (Fukawa et al., 2016). While that study posited a central role for tumor cells, our data support adipose as a major source for the FAs metabolized in muscle in cachexia. Moreover, preserving adipose tissue via pharmacological or genetic manipulation of lipolysis and lipogenesis protects muscle in other cachexia models (Das et al., 2011; Rohm et al., 2016; Tsoli et al., 2016), with the caveat that in all these studies multiple tissues other than adipose were also affected by the intervention.

Low skeletal muscle mass is predictive of mortality across diseases (Martin et al., 2013), which could be attributable to functional decline and respiratory or cardiac failure; however, the significance of fat loss in patients is less clear. The authors of a 2018 study discounted adipose loss as participating in PDAC mortality, based upon feeding pancreatic enzymes to mice with pancreatic cancer and upon cross-sectional body composition data in patients (Danai et al., 2018). Against that interpretation, however, are both clinical-correlative and mechanistic studies. Adipose inflammation is noted in patients with cancer cachexia (Camargo et al., 2015). Only fat was lost in patients with PDAC on neoadjuvant therapy, which is given early in the disease (Sandini et al., 2018). Fat loss in the absence of muscle loss has been observed in patients with PDAC (Sandini et al., 2018; Kays et al., 2018), and higher rates of adipose loss are associated with mortality in PDAC (Di Sebastiano et al., 2013). Furthermore, fat is lost more completely than muscle in PDAC. Patients with advanced/metastatic PDAC treated with FOLFIRINOX lost −29.5% ± 49.8% total adipose index versus −7.2% ± 13.3% skeletal muscle index (P = 0.026, unpaired *t* test with Welch’s correction; Kays et al., 2018). Myosteatosis, sarcopenia, and the combination were associated with reduced survival in one study of patients with resectable pancreatic and periampullary adenocarcinomas (Stretch et al., 2018) and myosteatosis associated with inflammation and reduced survival in another study of patients with unresectable pancreatic cancer or cholangiocarcinoma (Rollins et al., 2016). As well, obese patients with PDAC exhibit higher losses in weight, skeletal muscle, and adipose tissue along with poorer survival (Dalal et al., 2012). Even absent muscle wasting, low fat mass or “adipopenia” is associated with mortality in patients with diffuse large B cell lymphoma treated with immunotherapy (Camus et al., 2014) and in patients with heart failure (Melenovsky et al., 2013), among other conditions. Furthermore, longitudinal analysis of patients with advanced PDAC on FOLFIRINOX demonstrates equivalent mortality for patients with fat-only loss versus patients with both fat and muscle loss, with overall 10-mo reduced survival than patients without loss of either depot (Kays et al., 2018). Thus, the preponderance of clinical data points to an important role for adipose tissue in PDAC cachexia and mortality.

This study has limitations. Although our avatar data suggest a central role for IL-6 from malignant cells in cachexia, as also observed in C26 cachexia models (Petruzzelli et al., 2014), our results linking this to patient phenotypes is modest. We studied eight avatar lines, only three of which showed high IL-6; moreover, we are currently unable to associate expression of IL-6 in tumor cells to cachexia-related outcomes in patients with PDAC. Thus, further studies are required to strengthen this relationship. Soluble GP130 also modulates IL-6 trans-signaling (Wolf et al., 2016); however, this was not studied here. Presently, it is impossible to functionally dissociate classical signaling in muscle from muscle production of sIL6R for trans-signaling because genetic manipulation would affect both axes; thus, we must rely on cell models and inductive reasoning to arrive at our model. Furthermore, many factors upstream, downstream, and independent of IL-6 and IL6R clearly modulate the development and severity of cachexia in preclinical models (Baracos et al., 2018; Webster et al., 2020). Finally, we studied exclusively fat and muscle wasting mediated by tumor, although cachexia initiation and progression clearly also involve other tissues, including the brain, sympathetic nervous system, adrenals, gut, and liver (Grossberg et al., 2010; Olson et al., 2020). All of these questions require further investigation.

Clearly, cachexia is a complex systemic syndrome, and understanding changes in the macroenvironment of cancer is critical for designing effective treatment. The data provided here support targeted inhibition of sIL6R to treat PDAC cachexia. Preclinical studies document improved response to chemotherapy in mice with pancreatic cancer treated with anti-IL6R antibodies (Long et al., 2017); whether these mice also demonstrated reduced cachexia was not reported. Body composition and quality of life correlative studies in PACTO, NCT02767557, an ongoing trial of the IL6R inhibitor tocilizumab with gemcitabine and nab-paclitaxel in patients with advanced and metastatic PDAC, could provide level 1 evidence for this approach as anti-cachexia therapy (Chen et al., 2017). Additionally, our results add to the literature implicating fat loss as a potential driver of cachexia mortality and immunomodulation in cancer (Tsoli et al., 2016; Sun et al., 2020), supporting the need to monitor adipose in patients with cachexia-associated cancers (Ebadi and Mazurak, 2015). Finally, our studies suggest that strategies as diverse as targeting tumor cell production of IL-6, IL6R shedding from muscle and other tissues, adipose lipolysis, or lipid uptake by muscle might each show benefit in PDAC.

## Materials and methods

### IHC performed on human and murine tumors

Human tissues were collected with prior consent from both medical facility and individual patients as stated by US Biomax. Formalin-fixed human PDAC tumor arrays were obtained from US Biomax (PA1001a) and consisted of 100 core samples from 50 patients. 28 core samples consisting of pancreas tissue adjacent to tumor tissue or tumor samples having no reactivity for IL-6 were excluded. Removal of these specimens was justified since adjacent tissue is not actually healthy normal pancreas but rather tissue with a large degree of inflammation, and no reactivity for IL-6 in either stromal or epithelial compartments is typically an indication of poor tissue procurement. The final sample population was made up of 72 core samples and included 21 males and 15 females ranging in age from 40 to 78 yr. Tumor samples varied in stage from IB (maximum tumor diameter >4 cm) to IV (metastases in four or more regional lymph nodes) as defined by the eighth edition of the American Joint Committee on Cancer Cancer Staging Manual (Amin et al., 2017). Murine tumors were excised 17 d after injection and immediately fixed in 10% neutral buffered formalin (NBF) for 48 h. Tumors were then washed in PBS and stored in 70% ethanol at 4°C until analysis. Murine tumors were paraffin embedded, and cross sections were cut at 5 µm and mounted to microscope slides. Human and murine tumor sections were deparaffinized with two independent xylene washes (5 min each) and rehydrated using a series of diluted ethanol (100%, 95%, 70%) washes (5 min each) and rinsed twice in deionized water (dH_2_O; 5 min each).

Antigen retrieval was performed by incubating the rehydrated sections in a sodium citrate buffer (10 mM sodium citrate and 0.05% Tween 20, pH 6.0) at 95°C for 10 min and allowing them to cool to room temperature for 30 min. Sections were washed using dH_2_O, and endogenous peroxidase was quenched using 3% hydrogen peroxide (S25360; Thermo Fisher). Sections were incubated with blocking buffer that was composed of 8% FBS (SH3007103HI; Thermo Fisher) in PBS (21040CV; Corning) for 1 h and incubated overnight in a humid chamber at 4°C with an anti–IL-6 mAb against human IL-6 (ab6672; Abcam) diluted 1:400 in blocking buffer and mIL-6 (ab7737; Abcam) at 1:50 dilution in blocking buffer at 4°C overnight in a humid chamber.

Detection and visualization of the anti–IL-6 mAb was done using ImmPACT DAB Peroxidase (HRP) Substrate kit from Vector Laboratories (SK-4105). Sections were counterstained with hematoxylin for 1 min, dehydrated, and cleared using sequential incubations (3 min each) of dH_2_O, 70% ethanol, 95% ethanol, and 100% xylene, and then coverslips were applied using Cytoseal (831016; Thermo Fisher).

### Mouse PDAC models

Experiments that included mice were approved by and performed in accordance with the Indiana University School of Medicine Institutional Animal Care and Use Committee. Mice were group-housed in a barrier facility with ad libitum access to autoclaved food (Envigo) and sterile water, maintained on a 12-h light/dark cycle, and allowed to acclimate to the facility for 1 wk.

#### Implantation of human tumor fragments into mice

Patient consent was obtained before the collection and use of all human tissues in this study. Under Indiana University Institutional Review Board Protocol 1312105608, human tumor samples were collected from the operating room, placed in RPMI-1640 medium with 1% Pen/Strep, and transferred on ice to the laboratory. The tumor sample was divided into fragments of around 3 mm^3^ and dipped in matrigel before xenografting into male NSG: NOD.Cg-*Prkdcscid Il2rgtm1Wjl*/SzJ mice (In Vivo Therapeutics Core, Indiana University Simon Comprehensive Cancer Center), which lack mature T cells, B cells, and functional NK cells and are also deficient in cytokine signaling. All surgical procedures were performed using inhaled isoflurane followed by buprenorphine for 2 d per our Indiana University Institutional Animal Care and Use Committee–approved protocol. In the P0 generation, tumor fragments were transplanted through sewing of the fragment directly to the pancreas (orthotopic) and/or into the back under the skin (ectopic). For the former, the tail of the pancreas was exposed through a left lateral incision, and a single tumor fragment (3 mm^3^) was sutured to the pancreas using 6–0 nylon surgical sutures. Successful grafts were transplanted orthotopically to assess effects of tumor growth on cachexia and metastasis endpoints. P1 and P2 generations were created by repeating this procedure using 3-mm^3^ tumor pieces from the P0 and P1 mice, respectively. For cachexia studies, *n* = 7–9 mice were implanted with tumors from the P1 generation and kept in mixed cages with *n* = 3–7 sham control mice to serve as reference values for body mass and muscle size, given the long and varied time course of the different tumor lines. After tumor transplantation, the mice were monitored for tumor growth, body mass, body composition, and body condition scoring. All mice within one tumor group and their matched controls were euthanized when mean body condition score of the tumor-bearing mice reached ≥8 on a 11-point scale covering appearance, behavior, provoked behavior, clinical signs, and body habitus (Fig. S1; Paster et al., 2009). We report body mass loss as tumor-free body mass versus mean cohoused control body mass. Muscle wasting is expressed as fraction of muscle weight versus that of cohoused controls.

Euthanasia under general anesthesia (4% isoflurane) was done by exsanguination via cardiac puncture followed by cervical dislocation. Further details of these models will be published under separate cover.

#### Orthotopic implantation of KPC and KPC IL6^KO^ cells into mice

The anesthesia and surgical protocols have been described in detail within the generation of the PDX avatar methods. Briefly, 8-wk-old male C57BL/6J mice were anesthetized using 4% isoflurane and subcutaneously injected with buprenorphine at 0.5 mg/kg in 0.1 ml sterile PBS before surgery and then every 12 h from the first injection for 48 h. Mice were placed in lateral recumbency with the right side against the operating table, an incision was made in the abdomen, and the spleen and pancreas were retracted. The pancreas was injected with 5 × 10^4^ KPC or KPC IL6^KO^ cells in 50 µl growth media (GM) of cells using a 1-ml syringe with a 26G needle (329652; Becton, Dickinson and Co.) over a period of 30 s. The spleen and accompanying pancreas were gently laid back into the abdominal cavity using the ring tip forceps. The muscle wall was closed using suture, and the skin flap was closed using sterile wound clips.

### Euthanasia and tissue excision from animal models

All mice were euthanized when any group exhibited average body weight loss >10% or total fat mass <5% via EchoMRI. Euthanasia under general anesthesia (4% isoflurane) was done by exsanguination via cardiac puncture followed by cervical dislocation. Prior to euthanasia, blood was collected in EDTA tubes (366643; Becton, Dickinson and Co.) via cardiac puncture after anesthetizing the mice. The plasma was separated using centrifugation of the blood at 3,500 rpm at 4°C and stored at −80°C. Immediately following euthanasia, tissues were excised and weighed. Sections of tissues were placed into 2-ml cryogenic storage vials (82050–208; VWR) or snap frozen in liquid nitrogen. Additionally, sections of tumor were placed into tubes containing 10% NBF, and sections of epididymal fat were placed into tubes containing Bouin’s solution (23–005-69; Thermo Fisher) for 24 h. Fixed tumor and fat tissues were washed twice with PBS and transferred to new tubes containing 70% ethanol and stored at 4°C. Sections of the quadriceps muscle were mounted to cork discs, keeping the muscle fascicles perpendicular to the disc by using Tissue plus O.C.T. compound (23–730-571; Thermo Fisher). The muscle blocks were snap frozen by submerging the mounted muscle for 45 s in isopentane (2-methylbutane) (03551–4; Thermo Fisher) cooled to −150°C in a bath of liquid nitrogen and then stored at −80°C.

### IL-6 immunoassay and quantification of free FAs and glycerol

Prior to euthanasia, blood was collected in EDTA tubes (366643; Becton, Dickinson and Co.) via cardiac puncture after anesthetizing the mice. The plasma was separated using centrifugation of the blood at 3,500 rpm at 4°C and stored at −80°C. Immediately following euthanasia, tissues were excised, weighed, and snap frozen. Plasma IL-6 was measured using ELISA Quantikine kits for human (D6050) and mouse (MR600; R&D Systems). Plasma glycerol and FAs were measured using Glycerol and FA Quantitation Kits (MAK117 and MAK044; Sigma Aldrich).

### Cell culture models

C2C12 mouse myoblasts were from Dr. Paola Costelli (University of Turin, Turin, Italy). 3T3 fibroblasts were from Zen Bio (SP-LF-1). KPC 32908 tumor cells were from Dr. David Tuveson (Cold Spring Harbor Laboratory Cancer Center, Cold Spring Harbor, NY), who isolated them from a pancreatic tumor in a male LSL-KrasG12D:LSL-Trp53R172H:Pdx1-Cre (KPC) mouse on a C57BL/6 background (Hingorani et al., 2005), and we used these cells to generate the KPC IL6^KO^ cells (Research Resource Identifier: CVCL_UR56).

#### Generation of KPC and KPC IL6^KO^ tumor cell lines

Frozen KPC cells were resuscitated by thawing in a hot water bath at 37° C and then cultured in flasks with GM as described for C2C12 myoblasts. When cells reached 70% confluence, adherent cells were removed using trypsin (per C2C12 methods) and plated 0.3 × 10^6^ cells per well in two separate 6-well plates. Once cells were 70% confluent, Lipofectamine 3000 (L3000008; Invitrogen) was used to transfect one plate of KPC cells with a CRISPR/Cas9 plasmid (CRISPR06-1EA; Sigma Aldrich) encoded with a GFP and null gRNA. The additional plate was transfected with a CRISPR/Cas9 plasmid (MM0000468506; Sigma Aldrich) encoded with a GFP and a gRNA specifically targeting the *mIl6* gene. Cells were incubated for 48 h with the Lipofectamine and CRISPR plasmids. Cells were removed from the wells using trypsin and were sorted one GFP-positive cell per well into separate 96-well plates (167008; Thermo Fisher) using FACS. One 96-well plate contained cells transfected with the null plasmid, and the other plate contained cells transfected with the plasmid targeting *mIl6*. Single-cell cultures were maintained in GM and expanded into colonies over ∼4 wk. Colonies transfected with the plasmid targeting *mIl6* were screened for loss of *Il6* mRNA expression using RT-PCR. The selected clone with loss of *Il6* mRNA expression was designated KPC IL6^KO^. Colonies transfected with the null plasmid were screened using RT-PCR for *Il6* mRNA expression similar to that of the parental cell line (untransfected), and the selected control clone was designated KPC.

#### Proliferation assay of KPC cells

Comparison of in vitro proliferation between untransfected KPC cells (KPC-P), KPC cells transfected with a null CRISPR/Cas9 plasmid (KPC), and the mutant KPC IL6^KO^ cells was performed using the xCELLigence Real-Time Cell Analysis assay (ACEA Biosciences). Cells were plated in GM at 2,000 cells per well in triplicate in an Eplate 16 (ACEA Biosciences). Cell growth was measured each hour for 100 h.

#### C2C12 mouse myoblast cell culture and differentiation into myotubes

Frozen murine C2C12 myoblasts (2 × 10^6^ cells) were thawed in a 37°C water bath and then seeded in flasks (130191; Thermo Fisher) containing 20 ml of GM, which consisted of DMEM base media (10013CV; Corning), 10% FBS (SH30071.03; GE Healthcare Life Sciences), and 0.1% penicillin and streptomycin (15140122; Thermo Fisher) and maintained in a humidified incubator at 37°C with 5% CO_2_ until 70% confluent (∼2 d). The GM was aspirated, the cells were washed with PBS, and adherent cells were dislodged using 3 ml of 0.25% trypsin (25053CL; Corning). Cells were then seeded 0.3 × 10^6^ cells per well in 6-well plates (140685; Thermo Fisher) with GM and maintained at 37°C with 5% CO_2_ until 90% confluent. The GM was then aspirated, cells were washed with PBS, and 2 ml per well of differentiation media (DM) consisting of DMEM base media (10013CV; Corning), 2% horse serum (26050–088; Life Technologies), and 0.1% penicillin and streptomycin (15140122; Gibco) was added and cells maintained at 37°C with 5% CO_2_. The DM was replaced every 48 h, and C2C12 myoblasts were fully differentiated into myotubes after 5–6 d in DM.

#### Treatment of myotubes with KPC CM, IL-6, and IL-6 neutralizing antibody

Myotubes were maintained in DM and treated with either 30% KPC CM, 30% KPC IL6^KO^ CM or the combination of 30% KPC IL6^KO^ CM with recombinant mouse IL-6 (rIL-6, 300 pg/ml; 406-ML-005; R&D Systems), or 30% KPC IL6^KO^ with rIL-6 and IL-6 neutralizing antibody (2 mg/ml; 14–7061-85; Thermo Fisher), or 30% KPC CM with IL-6 neutralizing antibody for 48 h. After 48 h of treatment, myotubes were washed with PBS and fixed using ice-cold acetone/methanol (1:1) for 15 min at −20° C. Myotubes were then visualized with IF using an anti-myosin antibody (see IF methods), and myotube diameter was measured with Image J.

#### 3T3 fibroblast culture and differentiation into 3T3 adipocytes

Frozen 3T3 fibroblasts (0.5 × 10^6^) were thawed and seeded in flasks with preadipocyte media (PM-1-L1; Zen Bio) at 37°C with 5% CO_2_ in a humidified incubator until 70% confluent. Adherent cells were then dislodged using 0.25% trypsin, seeded 0.3 × 10^6^ cells per well in 6-well plates with preadipocyte media, and placed in the incubator. Cells were allowed to become 100% confluent, at which time cells were maintained at 100% confluence in preadipocyte media for an additional 48 h to ensure growth arrest, refreshing the preadipocyte media every 48 h. Cells were then washed with PBS, DM (DM-2-L1; Zen Bio) was added to the wells, and the cells were placed back into the incubator. Cells were cultured for 3 d in DM. Cells were then washed with PBS, adipocyte maintenance media (AM-1-L1; Zen Bio) was added to the wells, and the cells were placed back into the incubator. Cells were cultured in maintenance media for 10 d, refreshing the maintenance media every 48 h. At 10 d, fully differentiated 3T3 adipocytes were produced.

### RNA isolation, library preparation, sequencing, and quantitative PCR (qPCR)

#### RNA isolation from cells and whole tissue

RNA was isolated from ∼50 mg of snap-frozen tissue or from cells cultured in 6-well plates via the miRNeasy Mini Kit (217004; Qiagen). The concentration and quality of total RNA samples were first assessed using Agilent 2100 Bioanalyzer. An RNA Integrity Number of six or higher was required to pass the quality control. Isolated RNA from quadriceps and epididymal fat pads was taken from four animals per group (i.e., no tumor, KPC tumor, and KPC IL6^KO^ tumor mice) and subjected to next-generation sequencing.

#### cDNA library preparation and sequencing

For skeletal muscle, 100 ng of RNA per sample was used to prepare a single-indexed strand-specific cDNA library using the TruSeq Nano DNA Library Prep kit. For adipose tissue, 500 ng of RNA per sample was used to prepare a dual-indexed strand-specific cDNA library using the TruSeq Stranded mRNA Library Prep Kit (Illumina). The resulting libraries were assessed for quantity and size distribution using a Qubit and Agilent 2100 Bioanalyzer. 200 pM pooled RNA libraries was used per flow cell for clustering amplification on cBot using HiSeq 3000/4000 PE Cluster Kit and sequenced with 2 × 75–bp paired-end configuration on a HiSeq4000 (Illumina) using the HiSeq 3000/4000 PE SBS Kit. A Phred quality score (Q score) was used to measure the quality of sequencing. More than 95% of the sequencing reads reached Q30 (99.9% base call accuracy) for both muscle and adipose tissues.

The sequencing data were first assessed using FastQC (Babraham Bioinformatics), and then all sequenced libraries were mapped to the mm10 mouse genome using STAR RNA-seq aligner (Dobin et al., 2013). Uniquely mapped sequencing reads were assigned to the mm10 University of California Santa Cruz reference genome. The data were normalized using the trimmed mean of M values method. One muscle sample from the no tumor group was identified as an outlier and removed from further analyses. Differential expression analysis was performed using edgeR (McCarthy et al., 2012; Robinson et al., 2010), and the FDR was computed from P values using the Benjamini-Hochberg procedure. Differentially expressed genes were determined as having an FC of ≥1.5 and an FDR of ≤0.05. Ingenuity Pathway Analysis (Qiagen) and iPathway Guide (Advaita; Ahsan and Drăghici, 2017) were used for secondary analysis of RNA-seq results. Data are deposited into GEO datasets under series record accession no. GSE123310.

#### RNA isolation from tissue mononuclear cell fractions

Quadriceps and epididymal fat pads were excised immediately following euthanasia from no tumor control and KPC or KPC IL6^KO^ tumor–bearing mice and placed into cell culture GM. Tissues were then placed into Gentlemacs C tubes (130093237; Miltenyi) and dissociated using specialized kits for skeletal muscle (130098305; Miltenyi) or adipose tissue (130105808; Miltenyi) on a Gentlemacs Octo dissociator with Heaters (130096427; Miltenyi). Mononuclear cell suspensions were then filtered through a 40-µm strainer (22363547; Thermo Fisher) and resuspended in 5 ml of GM. Cells were then pelleted by centrifugation at 3,500 rpm for 10 min, GM was aspirated, and RNA was isolated as described previously in this section.

#### qPCR

For qPCR analyses, 100 ng of isolated RNA was reverse transcribed into cDNA using the VERSO cDNA Synthesis Kit (AB1453B; Thermo Fisher). Taqman Universal Master Mix II (4440038; Thermo Fisher) and gene-specific Taqman probes (*Il6* Mm00446190, *Il6ra* Mm01211444, *Trim63/Murf1* Mm01185221, *Fbxo32/Atrogin1* Mm00499523, and *Tbp* Mm01277042) were used to measure gene expression using cDNA in Light Cycler 480 96-well plates (04729692001; Roche) on a Light Cycler 96 (Roche). For each sample, TATA binding protein (*Tbp)* gene expression was used to normalize the expression of the target gene. Each well contained one PCR reaction, and reactions for each sample of cDNA were performed in triplicate for both target and control genes. Results from qPCR analyses were analyzed using the 2^-ΔΔCT^ method and reported as FC.

### Western blotting

Tissue protein lysates were made by homogenizing snap-frozen tissue in ice-cold radioimmunoprecipitation assay buffer (Harlow and Lane, 2006) using a Polytron PT 10/35 homogenizer with PCU 11 controller (Kinematica). Protein lysate concentration was measured using the Pierce BCA Protein Assay Kit (PI23228; Thermo Fisher). Protein lysate was then added 1:1 to 2× sample buffer (125 mM Tris, pH 6.8, 4% [wt/vol] SDS, 20% glycerol, 100 mM dithiothreitol, and 0.02% [wt/vol] bromophenol blue) and heated at 95°C for 5 min. Proteins were then separated via SDS-PAGE by loading 30 µg of protein from each sample into wells on a 4–15% Criterion TGX gel (5671084; Bio-Rad) in running buffer (25 mM Tris, 192 mM glycine, and 0.1% SDS) at 140 V for 1 h using a Power Pac HC (Bio-Rad). The proteins were transferred to 0.2-µm nitrocellulose membranes (1620233; Bio-Rad) in ice-cold transfer buffer (25 mM Tris, 192 mM glycine, and 20% [vol/vol] methanol, pH 8.3) at 100 V for 30 min. Membranes were blocked using Sea Block (37527; Thermo Fisher) for 1 h at room temperature on a shaker table. Proteins were detected using antigen-specific primary antibodies for ubiquitin (1:100; 3933; Cell Signaling), LC3B (1:100; L7543; Sigma Aldrich), Beclin-1 (1:100; 35955,; Cell Signaling), ATF4 (1:100; 11815; Cell Signaling), pAKT (1:100; 4060; Cell Signaling), AKT (1:100; 9272; Cell Signaling), p-4E-BP1 (1:100; 2855; Cell Signaling), 4E-BP1 (1:100; 9644; Cell Signaling), p-mTOR (1:100; 5536; Cell Signaling), mTOR (1:100; 2983; Cell Signaling), PGC1-α (1:100; ab54481; Abcam), PGC1-β (1:100; ab130741; Abcam), UCP2 (1:100; 89326; Cell Signaling), UCP3 (1:100; NBP2-24608; Novus Biologicals), and α-tubulin (1:100; 12G10; DSHB) diluted in Sea Block with 0.1% Tween 20 (BP337-500; Thermo Fisher) and incubated with the membranes overnight at 4°C. Membranes were then washed twice with PBS, and primary antibodies were visualized using florescent DyLight secondary antibodies (Cell Signaling) with specificity against the primary antibodies and imaged on an Odyssey CLx (LiCor). The quantification of target proteins was done by normalizing target protein expression to the loading control protein expression (α tubulin for muscle, β-actin for adipose tissue) specific to each membrane using Image Studio version 4.0 (LiCor). Normalized protein expression was then presented as FC versus no tumor–bearing mice.

### Muscle histology

For ORO staining, SDH reaction and IF were performed on muscle, snap-frozen mounted muscle blocks were serial sectioned (10-µm thickness) at −20°C on a Leica CM1860 cryostat, and muscle cross sections were mounted to Superfrost Plus microscope slides (12–550-15; Thermo Fisher).

#### ORO staining

ORO staining solution was prepared by dissolving 0.7 g of ORO (00625-25G; Sigma Aldrich) with 100 ml of 100% propylene glycol (398039; Sigma Aldrich) for 5 min at 100°C. The ORO staining was then maintained at 60°C until needed. Muscle cross sections were postfixed in 10% NBF for 10 min at room temperature. Sections were washed for 15 min in running tap water and then submerged in two separate baths of 100% propylene glycol for 5 min each. Sections were then incubated in ORO staining solution for 10 min with gentle shaking, washed in 85% propylene glycol for 3 min, and rinsed using distilled water for 3 min. Sections were counterstained with Harris Modified Hematoxylin (SH26-500D; Thermo Fisher), rinsed with running tap water for 10 min, and rinsed with distilled water for 3 min, and then counter slips were mounted using Prolong Gold Antifade Mountant (P36934; Life Technologies). ORO staining intensity was measured using established protocols (Mehlem et al., 2013).

#### SDH reaction

Glass Coplin jars were prewarmed to 37°C in an incubator before beginning the reaction. Succinic acid solution was made by adding 0.4 g succinic acid (6106–21-4; Acros Organics), 0.04 g nitroblue tetrazolium (N8129; Sigma Aldrich), and 0.001 g phenazine methosulfate (P9625; Sigma Aldrich) to 40 ml of 0.1 M Tris buffer and pH equilibrated to 7.01. The succinic acid solution was warmed to 37°C, and the muscle sections were submerged in the solution for 30 min, maintaining the temperature at 37°C in an incubator. Sections were then washed using a series of 30%, 60%, 90%, 60%, and 30% acetone (A18-1; Thermo Fisher) solutions for 5 s each and rinsed in distilled water. Coverslips were applied using Prolong Gold Antifade mounting media. SDH intensity was measured using color brightfield on the Lionheart LX Automated Microscope (Biotek).

### Visualization of C2C12 myotubes, muscle fiber dystrophin, and muscle IL6R expression

#### Visualization of myotubes

Myotubes were washed with PBS and fixed with 1:1 ice-cold acetone and methanol (A412-4; Thermo Fisher) at −20°C for 15 min. Myotubes were washed with PBS twice and blocked using blocking buffer (8% FBS in PBS) at room temperature for 1 h. Myotubes were then incubated with an anti-myosin primary mAb (MF 20; DSHB) at a dilution of 1:50 in blocking buffer overnight at 4°C in a humid chamber. Myotubes were washed with PBS and incubated with a florescent secondary antibody (A-11029; Thermo Fisher) for 1 h at room temperature in a humid chamber. Myotubes were washed with PBS and imaged, and myotube diameter was measured using Image J.

#### Muscle fiber dystrophin and IL6R

IF was performed on frozen muscle cross sections that were postfixed after sectioning in precooled acetone (−20°C) for 10 min and placed at room temperature for 30 min. Sections were washed twice in PBS and then blocked by incubating the sections with blocking buffer for 1 h at room temperature. The sections were washed with PBS and incubated with the primary antibodies against dystrophin (1:100; MANDRA11; DSHB), IL6R (1:500; ab83053; Abcam), and CD31 (1:500; ab24590; Abcam) diluted in blocking buffer overnight at 4°C in a humid chamber. Sections were washed, and the secondary antibody was applied for dystrophin (AlexaFluor 594 florescent secondary antibody; A-11032; Thermo Fisher), IL6R (AlexaFluor 594 florescent secondary antibody; A21209; Thermo Fisher), and CD31 (AlexaFluor 488 florescent secondary antibody; A11001; Thermo Fisher) at a dilution of 1:1,000 in blocking buffer for 1 h at room temperature. Sections were washed and incubated with DAPI (268298; EMD Millipore) for 1 min to visualize nuclei. Sections were washed, and coverslips were applied using Prolong Gold Antifade mounting media.

### Quantification of myotube diameter and muscle fiber cross-sectional area (CSA)

#### Myotube diameter

After visualization with IF, 20 random fields per well were taken at 20× magnification. The minimum diameters for approximately 10 myotubes per field were measured using Image J (National Institutes of Health) totaling ∼200 measurements for each well. The mean minimum myotube diameter was reported for each well, and a comparison of means (*n* = 3) for each condition was performed using ANOVA to determine statistical differences.

#### Myofiber CSA

Quadriceps muscle fibers were visualized using anti-dystrophin IF. Muscle fiber CSA was measured from 20 random fields taken across a single mid-belly quadriceps muscle section using an ImageJ macro that automatically contrasts the dystrophin IF and measures myofiber CSA, which we obtained from the Lieber laboratory (Mehlem et al., 2013; Minamoto et al., 2007). For each cross section, >300 myofibers were measured, and a mean was calculated. A comparison of means (*n* = 4) for each condition was done using ANOVA to determine statistical differences.

### Mouse adipose tissue IHC

Adipose tissue was excised and fixed in Bouin’s solution (2300569; Thermo Fisher) for 48 h, washed in PBS twice (10 min each), and stored in 70% ethanol at 4°C. Fixed adipose tissue was then paraffin embedded and sectioned at 5 µm and mounted to microscope slides. Deparaffinization, antigen retrieval, endogenous peroxidase neutralization, and blocking were performed as previously described for human and murine tumors. Sections were then incubated with an anti-murine IL-6 mAb (ab7737; Abcam) diluted 1:50 in blocking buffer overnight at 4°C in a humid chamber. Detection of the primary mAb, counter staining, clearing, and coverslip application were performed as described for the human and murine tumors.

### KPC CM treatment of adipocytes and myotubes

KPC cells were cultured in GM in flasks until becoming 100% confluent. At that time, the GM was refreshed, and 100% confluent KPC cells were maintained for an additional 24 h. After 24 h, the CM was collected and centrifuged at 3,500 rpm to pellet unwanted cellular debris, and the supernatant was collected.

C2C12 myoblasts and 3T3 fibroblasts were cultured separately in 6-well plates and differentiated into myotubes and adipocytes, respectively (see in vitro cell culture). Myotubes were maintained in DF, and adipocytes were maintained in adipocyte maintenance media and treated in triplicate with either 30% GM (control) or 30% KPC CM per well. For myotubes, the treatment media was aspirated, cells were washed, and RNA from myotubes was isolated at 12 h, 48 h, and 96 h after treatment. For 3T3 adipocytes, RNA was isolated at 1 h and 3 h after treatment in the same collection method used for the myotubes. The time points selected for 3T3 adipocytes were selected from established protocols for the measurement of lipolysis (Schweiger et al., 2014). Myotube and adipocyte expression of *Il6* and *Il6r* mRNA at each time point was measured via qPCR using probes for *Il6* (Mm00446190; Applied Biosciences) and *Il6r* (Mm01211444; Applied Biosciences).

### KPC, adipocyte, and myotube media-swap studies

Mature adipocytes were treated with 30% KPC CM for 1 h, the KPC CM was removed, adipocytes were washed with PBS, and adipocyte maintenance medium was applied to collect the adipocyte secretome in response to KPC CM stimulation. This adipocyte CM was then harvested at 1 h and 3 h after KPC CM stimulation. Myotubes were then treated with 30% adipocyte CM for 48 h, and myotube diameter was measured.

Myotubes were treated with 30% KPC CM for 12 h, 48 h, and 96 h, and the myotube CM was collected at each of these time points. Adipocytes were then treated with 30% C2C12 myotube CM from each time point (i.e., 12 h, 48 h, 96 h) for 1 h, C2C12 CM was then removed, adipocytes were washed with PBS, and adipocyte maintenance medium was applied. Adipocyte CM was harvested at 1 h and 3 h after C2C12 CM stimulation and measured for glycerol content as a marker for lipolysis.

### Treatment of myotubes with IL-6, IL6R, and IL-6 and palmitate

Myotubes were maintained in DF and treated with either PBS (control), recombinant mouse IL-6 (300 pg/ml; 406-ML-005; R&D Systems), recombinant mouse IL6R (25 ng/ml; P9767-5G; Sigma Aldrich), and the combination of IL-6 (300 pg/ml) and IL6R (25 ng/ml) for 48 h. After 48 h, myotubes were fixed, and mean minimum diameters were measured as previously described herein.

Separately, myotubes were maintained in DF and treated with either PBS (control), recombinant mouse IL-6 (20 ng/ml; 406-ML-005; R&D Systems), palmitate (0.5 mM; P9767-5G; Sigma Aldrich) conjugated to BSA in a 5:1 molar ratio, or the combination of IL-6 (20 ng/ml) and palmitate (0.5 mM) for 48 h. After 48 h, myotubes were fixed, and mean minimum diameters were measured as previously described herein.

### Treatment of adipocytes with IL-6 and IL6R

Adipocytes were maintained in adipocyte maintenance media and treated with PBS (control), recombinant mouse IL-6 (300 pg/ml; 406-ML-005; R&D Systems), recombinant mouse IL6R (25 ng/ml; P9767-5G; Sigma Aldrich), or the combination of IL-6 (300 pg/ml) and IL6R (25 ng/ml) for 1 h. After 1 h, adipocytes were washed, and adipocyte maintenance medium was refreshed without the presence of IL-6 or IL6R. Adipocyte medium was then collected 1 and 3 h later for quantification of media glycerol concentrations to measure lipolysis, as described in these methods previously.

### Statistical analyses

Unpaired two-tailed Student’s *t* test was used for the comparison of means between two datasets, while one-way ANOVA with the Tukey’s post hoc test was used for comparison of the means among three or more datasets. The differences between group means were considered statistically different when P < 0.05. Data are presented as mean ± SD or SEM as in figure legends. All statistical analyses were performed using Graph Pad Prism versions 7.0–9.0.

### Online supplemental material

Fig. S1 presents a table outlining the body condition scoring used for experimental mice (related to Fig. 3) and shows the heterogeneous expression of *Il6* mRNA in human PDAC cell lines (related to Fig. 1). Fig. S2 presents the CRISPR plasmid map targeting the murine *Il6 *gene and shows the DNA sequence for the mutation induced in the murine *Il6 *gene after transfection with the CRISPR plasmid (related to Fig. 2). Fig. S3 shows changes in mouse body, carcass, liver, and spleen weights (related to Fig. 3), shows no correlations between tumor weight and gastrocnemius, tibialis, or epididymal fat pad weights (related to Fig. 3), and provides the scoring criteria used for determining mouse tumor grades (related to Fig. 3). Fig. S4 lists differentially regulated pathways in epididymal fat identified from RNA-seq data using Ingenuity Pathway Analysis (related to Fig. 6) and shows a differentially regulated cell population in epididymal fat identified using deconvolution analysis of the RNA-seq data (related to Fig. 6). Fig. S5 displays mean Ct values for *Il6* and *Il6r* mRNA expression in tissues analyzed using qPCR (related to Fig. 7) and shows IL-6 and IL6R protein expression in various tissues from the GTex Portal Database (related to Fig. 7).
